# Exploring the Comorbidity Mechanism among Functional Diarrhea, Irritable Bowel Syndrome with Predominant Diarrhea, and Functional Dyspepsia through the Gut-brain Axis and the Pharmacological Mechanisms of Shen-Ling-Bai-Zhu-San Therapy

**DOI:** 10.2174/0113862073329698250102120037

**Published:** 2025-04-18

**Authors:** Chun-feng Mei, Li-li Han, Hong Xue

**Affiliations:** 1 Digestive Laboratory of Traditional Chinese Medicine Research Institute of Spleen and Stomach Diseases, Xi-yuan Hospital, China Academy of Chinese Medical Sciences, Beijing, 100091, China

**Keywords:** Shen-Ling-Bai-Zhu-San, functional gastrointestinal diseases, gut-brain axis, network pharmacology, molecular docking, molecular dynamics simulations, bio-layer interferometry

## Abstract

**Background:**

Functional Diarrhea (FDr), Irritable Bowel Syndrome with predominant diarrhea (IBS-D), and Functional Dyspepsia (FD) are common functional gastrointestinal disorders (FGIDs) with significant impacts on quality of life. While the gut-brain axis and key regulators like 5-hydroxytryptamine (5-HT), dopamine (DA), and butyrate are known to play crucial roles in these conditions, the mechanisms underlying their comorbidities remain unclear. Shen-Ling-Bai-Zhu-San (SLBZS), a traditional herbal formula, is effective in treating FGIDs. Still, the specific components and mechanisms mediating its therapeutic effects via the gut-brain axis are not well understood.

**Methods:**

This study identified molecular links among FDr, IBS-D, and FD from the gut-brain axis using integrated biological information. The pharmacological mechanisms of SLBZS were explored through network pharmacology, molecular docking, molecular dynamics simulations, and *in vitro* bio-layer interferometry (BLI) validation.

**Results:**

A total of 328 common targets were identified among FDr, IBS-D, and FD, with 22 hub genes in the protein-protein interaction (PPI) network associated with 5-HT/DA/butyrate pathways. Virtual screening revealed seven key targets (AKT1, CASP3, VEGFA, INS, CTNNB1, PTGS2, and IL1B) and 14 bioactive components (e.g., diosgenin and luteolin) from SLBZS. Molecular docking indicated strong binding affinities between key components and targets, while molecular dynamics simulations confirmed stable interactions, particularly between PTGS2 and diosgenin/luteolin. BLI experiments further validated the strong binding affinity of PTGS2 for diosgenin.

**Conclusion:**

This study elucidates potential targets shared by FDr, IBS-D, and FD through the gut-brain axis, highlighting diosgenin's interaction with PTGS2 as a potential mechanism of SLBZS's therapeutic effects. These findings provide valuable insights into the pharmacological mechanisms of SLBZS in treating FGIDs and suggest new avenues for targeted therapies.

## 1 INTRODUCTION

FDr is defined as the continuous or repeated excretion of pasty or liquid salts with a global prevalence of 4.7% [1, 2]. IBS-D is a symptom-based disorder characterized by abdominal pain and diarrhea, affecting approximately 31.5% of individuals with IBS [3, 4]. FD refers to a complex of symptoms referable to the gastroduodenal region of the upper gastrointestinal tract with an approximately 16% prevalence [5]. These disorders represent the most researched and salient disorders among FGIDs, which are characterized by the absence of organic disorders [1]. FDr and IBS-D share similar symptoms and treatment approaches, with the European Society of Gastroenterology acknowledging a potential overlap between the two conditions [6–8]. Studies indicate that 29% to 70.5% of IBS patients also suffer from overlapping FD, while 14% to 31% of FD patients are diagnosed with IBS [9–12]. The overlap rate between FDr and FD is reported to be 1.5% [13]. Notably, significant overlapping FGIDs result in higher disease severity, lower quality of life, and more social burden than individuals who have a single FGID diagnosis [1, 9, 14].

Recent research indicates that these conditions share common physiopathological mechanisms, including disorders of dysregulation of the gut-brain axis, visceral hypersensitivity, abnormal motility, microbiome imbalance, immune dysregulation, inflammation, and barrier dysfunction [15–17]. The gut-brain axis (GBA) is a bidirectional communication system between the brain and the gut. Many neurotransmitters and signaling molecules such as glutamate, serotonin (5-HT), dopamine, acetylcholine, neuropeptides, inflammatory mediators, gut microbiota, and their metabolic products [18] are involved in the gut-brain axis. 5-HT, DA, and short-chain fatty acids (intestinal microbial metabolites) are generally acknowledged as three important molecules in the gut-brain axis [19, 20]. Therefore, our next research steps will focus on these three molecules.

5-HT and DA are critical for intestinal visceral sensation, and their metabolic abnormalities significantly contribute to the pathophysiological mechanisms of FGIDs [19, 21–24]. Certain neuromodulators related to 5-HT and DA can regulate gastrointestinal sensation and motor function, which have been utilized in clinical treatment for FGIDs [25]. Short-chain fatty acids (SCFAs), produced primarily through bacterial fermentation of dietary fiber, can be directly or indirectly involved in microbiota-gut-brain crosstalk [26]. Butyrate, one of the most important SCFAs, maintains intestinal homeostasis by protecting the integrity of the intestinal epithelial barrier and regulating the function and differentiation of intestinal immune cells through inhibition of histone deacetylases or activation of G protein-coupled receptors [27]. Moreover, butyrate crosses the blood-brain barrier, exerting acute and chronic effects on the brain structure and function [26, 28]. We hypothesize that 5-HT, DA, and butyrate contribute to the overlap among FDr, IBS-D, and FD through the gut-brain axis; however, the specific molecular links remain to be clarified.

Till now, available treatments for these three diseases have included prokinetics, antispasmodics, and peripherally acting neuromodulators, as well as lifestyle modifications and dietary and gut microbiota manipulation. [15, 29] However, due to the overlap among these diseases, the clinical efficacy has not met the expectations of patients and physicians. Therefore, for better treatment of these diseases, the identification of more drug candidates is urgently needed, and TCM may provide some new options.

Shen-Ling-Bai-Zhu-San (SLBZS), a famous Traditional Chinese medicine (TCM) formula from Tai Ping Hui Min He Ji Ju Fang, consists of eleven herbs: Ren-Shen (RS), Bai-Zhu (BZ), Fu-Ling (FL), Shan-Yao (SY), Lian-Zi (LZ), Bai-Bian-Dou (BBD), Yi-Yi-Ren (YYR), Sha-Ren (SR), Jie-Geng (JG), Gan-Cao (GC) and Da-Zao (DZ). Qiu *et al.* [30] and Ou *et al.* [31] conducted clinical trials demonstrating that Shen-Ling-Bai-Zhu-San (SLBZS) effectively treats FUNCTIONAL DIARRHEA (FDr) and is safer than other traditional drugs. Zhang *et al.* found that SLBZS significantly reduced the clinical symptoms of IBS-D patients when combined with the standard treatment of a control group [32]. Additionally, a meta-analysis of 12 studies involving 1634 patients with functional dyspepsia suggested that SLBZS may offer potential efficacy for this condition [33]. The effectiveness of SLBZS is largely due to its multifactorial approach, the actions of its herbal components, and its good safety profile.

Previous research has shown that SLBZS may exert therapeutic effects on FGIDs by reducing the expression levels of 5-HT [34, 35]. It has been found that RS, as a sovereign of SLBZS, can modulate dopamine release or the pain sensitivity of dopamine D2 receptors [36]. Our recent study discovered that SLBZS alleviated lactose-induced diarrhea by modulating the gut microbiota and enhancing butyrate production [37]. These findings suggest that SLBZS can treat FGID through the regulation of 5-HT, DA, and butyrate. However, the underlying mechanisms of these potential treatments require further elucidation.

Based on systems biology, polypharmacology, and computational biology, network pharmacology is a discipline that explores the correlation between drugs and diseases holistically and systematically [38, 39]. The application of network pharmacology is an investigation into the mechanism of multiple components, multiple targets, and multiple pathways in the treatment of unspecified diseases [40]. Furthermore, integrating network pharmacology with experimental verification offers an alternative approach to identifying potential drug targets.

This study aimed to uncover the mechanisms underlying the comorbidity of FDr, IBS-D, and FD, as well as how SLBZS exerts therapeutic effects on these conditions via the brain-gut axis by combining network analysis, computer validation, and *in vitro* experimental validation.

## 2 MATERIALS AND METHODS

### 2.1 Screening for Targets of FDr, IBS-D, and FD

Disease targets are sourced from gene-centric databases covering genomics, transcriptomics, proteomics, etc., such as GeneCards (https://www.genecards.org/) [41], OMIM (https://omim.org/) [42] and DisGeNET (https://www.disgenet.org/) [43]. According to the “relevance score” in GeneCards, data were filtered by the median. After removing the repetitive targets, the targets of FDr, IBS-D, and FD were intersected using the online software Venny 2.1.0 to obtain common targets and create a Venn diagram.

### 2.2 Gene Ontology (GO) and Kyoto Encyclopedia of Genes and Genomes (KEGG) Pathway Enrichment Analysis

To explore the common gene functions, Metascape [44], a gene annotation and analysis database was utilized to perform GO and KEGG pathway enrichment analysis of the common targets. The species was restricted to “Homo sapiens”. GO molecular functions (MF), GO biological processes (BP), GO cellular components (CC), and KEGG pathway enrichment analysis were performed. Visualization of the results was carried out using R 4.2.1 software.

### 2.3 Protein‒Protein Interaction (PPI) Network Construction

We chose 5-HT, DA, and butyrate as subjects further to explore the possible mechanisms of the gut-brain axis. Targets associated with 5-HT, DA, and butyrate were retrieved from the National Center for Biotechnology Information (NCBI) (https://www.ncbi.nlm.nih.gov/), which offers a comprehensive array of biological information and data resources. To obtain the PPI data, the common targets of 5-HT/DA/butyrate, along with FDr, IBS-D and FD were imported into STRING [45] and integrated all known and predicted associations between proteins, including both physical interactions and functional associations [45]. The resulting PPI data were exported in TSV format and imported into Cytoscape 3.9.1 software [46] to construct the PPI network. Furthermore, we performed additional analysis on the PPI network by calculating MCC by cytoHUBBA to get hub genes [47].

### 2.4 Active Ingredients and Targets of SLBZS

The active components of SLBZS were identified and collected from the Traditional Chinese Medicine Systems Pharmacology Database and Analysis Platform (TCMSP) [48], the Encyclopaedia of Traditional Chinese Medicine (ETCM) [49], and the published literature. Selecting “Human” in “Reviewed (Swiss-Prot),” a universal Protein Resource Knowledge base (Uniprot) [50] was applied to unify the standard nomenclature.

### 2.5 Drug Ingredients-5-HT/ DA/ Butyrate-Targets Network Construction and Analysis

The first step in this process was to construct a drug-Components-(H-D-B)-target network using Cytoscape 3.9.1. According to the network, the key targets and components were prepared and ranked by degree and betweenness in CytoNCA. For further screening, we introduced ADME parameters and performed specific analysis for each drug based on reported literature, oral bioavailability (OB) and drug-likeness (DL). OB is an important indicator that determines whether orally administered drugs could overcome several barriers, and DL is a qualitative concept used in drug design for an estimate of how “drug-like” a prospective compound is, which is used to distinguish drugs from nondrugs. The parameter of 30% was used to exclude the components with low oral availability according to the TCMSP parameter recommendation and literature. The threshold of DL is determined by the fact that the average DL index in the Drugbank is 0.18, which is used as a selection criterion for the ‘drug-like’ components in traditional Chinese herbs.

### 2.6 Enrichment Analysis of Key Targets

To further explore the action mechanisms of SLBZS for the treatment of FDr, IBS-D and FD based on the gut-brain axis, we obtained the intersection genes of SLBZS-5-HT/DA/butyrate-diseases. The same steps as in Method 2.2 were used to perform enrichment analysis on the common targets using STRING.

### 2.7 Molecular Docking

Molecular docking is a well-established structure-based computational method used to identify novel compounds with therapeutic potential and predict ligand-target interactions at the molecular level. Ligand files of top key components were prepared by downloading from PubChem (https://pubchem.ncbi.nlm.nih.gov) [51], and energy minimization was performed through Chem3D. Then, we hydrogenated the ligand, checked and adjusted all torsions of the ligand, and then converted the mol2 format to pdbqt format using AutoDock 1.5.7. The selection criteria of receptor proteins are that the species is human, has the best resolution, and binds to the original ligand. The pdb format files of top key targets were downloaded from the RCSB Protein Data Bank, [52] along with the water, ligands and symmetrically repeated chain were removed using PyMoL. Then, we adopted the operation of hydrogen addition, calculation of electrons, and assigning AD4 type by importing the target pdb file into AutoDock 1.5.7, after which the pdbqt file was exported. We constructed a docking grid box large enough to cover each receptor, which contained all possible docking pockets. Using Vina [53], the ligands of components and receptors of targets were docked, and the minimum affinity was obtained. The "pheatmap" package was used to create a heatmap of the minimum affinity in R 4.2.1. PyMoL and Ligplot software were used to visualize molecular docking results.

### 2.8 Molecule Dynamic Simulation

Molecular dynamic (MD) simulation is a computational method that can evaluate the dynamic characteristics and stability of the component-target protein complexes. The molecular docking results were used as the initial conformation for MD simulations. Gromacs 2022 software was used to run MD simulations. Select CHARMM 36 force field parameters and the default water model (CHARMM-modified TIP3P) to prepare the protein topology. Upon generation of the ligand topology with CGenFF, the complexes were built. After completing the NVT and NPT simulations, the system is now well-equilibrated at the desired temperature and pressure. Finally, MD simulations were run for 100 ns. The root means square deviation (RMSD), root mean square fluctuation (RMSF), snapshots of the MD trajectories, center-of-mass distance, and free energy landscape were performed.

### 2.9 In Vitro Validation

BLI is a label-free technique that converts light interference signals occurring on the surface of BLI biosensors into real-time response signals [54]. By monitoring the molecular binding process in real time, the system determines the association and dissociation constants, and through fitting calculations, it analyzes and obtains information on affinity and concentration [54]. PTGS2 was procured from SinoBiological, while diosgenin was obtained from MedChemExpress. The selected target was further validated using BLI assay on Octet R8. In brief, all runs were conducted with a shaking speed of 1000 rpm and a plate temperature of 30°C. PTGS2-His-tag protein at 50 μg/mL was immobilized on Octet NiNTA biosensors (Sartorius). Background binding control utilized the same sensors incubated in PBS kinetics buffer without proteins. Diosgenin at various concentrations was diluted with PBS containing 0.02% Tween-20 and 10% DMSO and added to a black polypropylene 96-well microplate (Greiner Bio-one) with PBS filling the remainder of the wells. After washing for 60 seconds and establishing a baseline with PBS containing 0.02% Tween-20 and 10% DMSO, biosensors were immersed in wells containing different concentrations of diosgenin to allow for association of the drug (120–180 seconds). Subsequently, a dissociation step was performed (120–180 seconds). All data were analyzed using Octet data analysis software version 13.0, with KD, Kon, Koff, and R2 values reported as the evaluation indices.

## 3 RESULTS

### 3.1 Identification and Enrichment Analysis of Overlapping Targets of FDr, IBS-D, and FD Based on the Gut-Brain Axis

The related targets of FDr, IBS-D, and FD were acquired from the GeneCards, OMIM, and DisGeNET databases. After removing duplicate results, we identified 1446 targets for FDr, 1294 targets for IBS-D, and 883 targets for FD. Using Venny 2.1.0 software to find the intersections of these disease-related targets, we obtained 328 common targets, as shown in the Venn diagrams in Fig. (**1A**). To further investigate the function of common targets, we performed GO and KEGG pathway enrichment analyses using Metascape database. Restricted to a *P* value less than 0.05, a total of 197 BP terms, 193 CC terms, 305 MF terms, and 207 KEGG enrichment pathways were acquired. Parts of terms with a *P* value < 0.01, a minimum count of 3, and an enrichment factor > 1.5 are shown in Supplementary Table **1**. The GOMF analysis for the three diseases' association was relevant to serotonin binding and fatty acid binding (Fig. **1B**).

### 3.2 Construction of PPI Networks and Identification of Hub Genes Associated with Common Targets in the Gut-Brain Axis

Given the importance of 5-HT, DA, and butyrate in these three diseases, we aimed to predict common gut-brain-related targets and construct the PPI network. We obtained 117 genes for 5-HT, 679 genes for DA, and 133 genes for butyrate from NCBI. By intersecting these genes with the 328 common targets of the three diseases, we identified 25 common genes for 5-HT-FDr-IBS-D-FD (diseases), 51 genes for DA-diseases, and 39 genes for butyrate-diseases (Table **1**). To investigate the relationships among these targets, the PPI data were obtained using the STRING database with parameters filtered by “Homo sapiens” and “medium confidence (0.400)”. After hiding disconnected nodes, the 5-HT-diseases PPI network contained 25 nodes and 128 edges, the DA-diseases PPI network contained 51 nodes and 472 edges, and the butyrate-diseases PPI network contained 39 nodes and 453 edges. The PPI network files were imported into Cytoscape 3.9.1 for visualization, and the results were displayed in Fig. (**2A**-**C**). Using cytoHUBBA topology analysis, MTOR, IFNG, IL4, TLR4, AKT1, PTEN, BDNF, NOS3, FOXP3, and TLR2 were considered as hub genes in the 5-HT-diseases network (Fig. **2D**), AKT1, IL6, VEGFA, INS, BDNF, LEP, CTNNB1, CASP3, IL10, and PTGS2 were identified as the hub genes in the DA-diseases PPI networks (Fig. **2E**), and TP53, CTNNB1, HIF1A, IL1B, HRAS, PTGS2, IL6, TGFB1, MYC and KRAS in butyrate-diseases PPI network (Fig. **2F**).

### 3.3 Bioactive Ingredients and Targets of SLBZS

A total of 1091 active components of SLBZS were initially retrieved. After removing duplicates, 987 unique components remained, distributed as follows: 190 from RS, 55 from BZ, 34 from FL, 71 from SY, 9 from LZ, 38 from YYR, 14 from BBD, 165 from SR, 102 from JG, 280 from GC, and 133 from DZ (Table **2**). In total, 8946 potential targets were identified in TCMSP. After eliminating duplicates and converting the potential targets into gene names using UniProt, we obtained 616 component-related targets (Supplementary Table **2**).

### 3.4 Drug-ingredients-5-HT/DA/butyrate-Diseases Hub Gene Network Construction

According to the above results, displaying the important role of 5-HT/DA/butyrate in three diseases, and the previous study showing SLBZS’s regulation of the gut-brain axis [34], we speculated that SLBZS could improve the symptoms of three diseases through 5-HT/DA/butyrate. Consequently, drug-component-5-HT/DA/butyrate-hub gene networks were constructed (Fig. **3**). Using the CytoNCA plugin, nodes with degree and betweenness values greater than or equal to twice the median were considered key components and targets. In the 5-HT network, NOS3, AKT1, PTEN, IFNG, BDNF, and IL4 were identified as key targets, and quercetin, kaempferol, luteolin, formononetin, diosgenin, beta-carotene, and ginsenoside Rf were identified as key components (Fig. **3A**). In the DA network, key targets were PTGS2, CASP3, IL10, IL6, AKT1, VEGFA, INS, BDNF, and CTNNB1, while key components were quercetin, beta-carotene, kaempferol, beta-sitosterol, luteolin, stigmasterol, pronuciferine, diosgenin, naringenin, acacetin, ginsenoside Rh2, and fumarine (Fig. **3B**). For the butyrate network, PTGS2, IL6, IL1B, and TP53 were key targets, and quercetin, stigmasterol, beta-sitosterol, and beta-carotene were key components (Fig. **3C**).

### 3.5 GO and KEGG Analysis of 5-HT, DA and Butyrate (H-D-B) related Therapeutic Targets and SLBZS-key Ingredients- (H-D-B) Therapeutic Target-(H-D-B)- Therapeutic Pathway Network Analysis

To illuminate the complex mechanisms, we performed GO and KEGG enrichment analysis on the 15 therapeutic targets obtained after deduplication of the above three networks, resulting in 559 terms (Supplementary Tables **1-3**). According to GOBP and KEGG enrichment analysis, pathways such as serotonergic synapse, dopaminergic neuron differentiation, negative regulation of fatty acid metabolic process, response to fatty acid, regulation of fatty acid transport and regulation of fatty acid metabolic process which were common among the three diseases, also appeared in the enrichment pathways of SLBZS therapeutic targets (Fig. **4A**). These findings confirmed that the key components of SLBZS exert therapeutic effects in the three diseases by regulating 5-HT, DA, and butyrate. Furthermore, to elucidate the relationships among key components, therapeutic targets, and pathways, we constructed a network as shown in Fig. (**4B**). The network consisted of 9 drugs, 14 components, 7 targets (AKT1, CASP3, VEGFA, INS, CTNNB1, PTGS2 and IL1B), 6 enrichment terms. It suggests that the action mechanism of SLBZS may act through 14 key components targeting 7 key genes via 5-HT, DA, and butyrate-related pathways. Details of the 7 key targets and 14 components are presented in Tables **3** and **4**.

### 3.6 Verification by Molecular Docking

To further evaluate the interactions between the above-identified 14 components and 7 targets enriched in 5-HT, DA and butyrate-related pathways, we performed molecular docking to calculate the binding affinities. All affinities were less than -5.8 kcal/mol, suggesting good binding ability between key targets and components (Fig. **4C**). Among them, the PTGS2-diosgenin interaction exhibited the lowest affinity (-11.7 kcal/mol). Furthermore, the binding interactions of PTGS2- diosgenin and PTGS2-luteolin were visualized using Ligplot and PyMoL (Fig. **5A**). Different numbers of hydrogen bonds and hydrophobic interactions docked the components and targets. We observed one hydrogen bond with Asn537(A) and 14 hydrophobic interactions between PTGS2 and diosgenin ,6 hydrogen bonds with Asp125(B), Arg44(B), and Cys47(B), and 8 hydrophobic interactions between PTGS2 and luteolin.

### 3.7 Molecular Dynamic Simulation

Since the absolute motion of proteins and small ligands is not considered in the molecular docking process, we further verified the stability of the docking results PTGS2- diosgenin, PTGS2- luteolin with the lowest docking energy through MD simulation. The RMSD reflects the fluctuation in protein conformation, while the ligand RMSD allows us to understand how stable the ligand is over the simulation time. The average RMSD values were 1.95 Å (PTGS2- diosgenin backbone), 0.70 Å (diosgenin), 2.66 Å (PTGS2- luteolin backbone), 1.19 Å (luteolin), respectively. As shown in Fig. (**5B**), after initial fluctuations, all molecules reached dynamic equilibrium with minor fluctuations, gradually stabilizing. The RMSF reflects the fluctuation of the amino acid residues of protein. The average RMSF values were 1.60 Å (PTGS2- diosgenin) and 1.72 Å (PTGS2- luteolin) (Fig. **5C**). The low fluctuation of the complex indicated great stability. Superimposed snapshots of the MD trajectories and binding poses at the active binding pocket of PTGS2 at different time points (0 ns, 10 ns, 20 ns, 40 ns, 60 ns, 80 ns, and 100 ns) were shown in Fig. (**6A**). These snapshots were then aligned to identify the location of the docked compounds and assess the dynamic regions of the protein. During the 100 ns MD simulation, the receptor and ligand endeavored to achieve stability within the active binding pocket by seeking the most stable binding pose. During the 100 MD simulation, diosgenin maintained distances of approximately 2-4 nm from PTGS2. Similarly, the distance between luteolin and PTGS2 fluctuated within the range of approximately 2-4.5 nm, respectively (Fig. **6B**). Free energy landscapes showed the PTGS2 and diosgenin-bound complex stability in terms of Gibbs free energy, which was analyzed by PCA. The complex systems contained three main basins indicating a relatively stable state and one of the stable 3D protein structures was shown in Fig. (**6C**). The MD system showed a relatively great state which could exist stably in terms of RMSD, RMSF, and free energy landscapes. Therefore, diosgenin and luteolin, the key components of SLBZS, play an important role in improving the comorbidity among FDr, IBS-D, and FD by acting on PTGS2.

### 3.8 BLI-Initiated Verification Demonstrates that PTGS2 Exhibits a Strong Affinity for Diosgenin

To verify the binding affinity of diosgenin to the PTGS2 protein, which showed better MD results, we conducted BLI analysis using diosgenin at concentrations of 1.875, 3.75, 7.5, and 15 μM. The analysis was performed with His-tagged human recombinant PTGS2 immobilized on a nickel-nitrilotriacetic acid probe. The results revealed a dose-dependent binding of diosgenin to PTGS2, with a calculated KD value of 1.3×10^-5^ M, a kon of 3.51×10^-3^ (1/ms), and a kdis of 4.58×10^-2^ (1/s) (Fig. **6D**). Steady-state analysis indicated a coefficient of determination of 0.9966, underscoring the accuracy and reproducibility of the binding affinity measurements (Fig. **6E**). These findings confirmed that diosgenin binds to PTGS2 in a concentration-dependent manner, with higher concentrations of diosgenin resulting in stronger binding interactions.

## 4 DISCUSSION

In this study, we systematically investigated the comorbid molecular mechanisms of FDr, IBS-D, and FD, as well as the bioactive compounds of SLBZS and their potential therapeutic mechanisms, using a combination of network pharmacology, computer simulations, and experimental validation.

We first searched public databases to obtain the relevant targets for FDr, IBS-D, and FD. A total of 328 common targets and 902 enrichment analysis terms involved in the progression of these three diseases were identified. The PPI networks of 5-HT/DA/butyrate-diseases revealed 22 hub genes including VEGFA, INS, IL6, TNF, AKT1, STAT3, IFNG, TP53, EGF, SRC, BDNF, LEP, CTNNB1, CASP3, IL10, PTGS2, HIF1A, IL1B, HRAS, TGFB1, MYC, and KRAS which played crucial roles in the shared molecular mechanisms.

Previous works have demonstrated that SLBZS exerts therapeutic effects against FGID by regulating the content of 5-HT, SCFAs, and DA, suggesting that SLBZS has an effect on the gut-brain axis [55]. However, the specific active component remains unknown. Our study identified 987 unique components and 616 different corresponding targets of SLBZS, which is different from modern medicine with single-target characteristics. Seven targets enriched in the serotonergic synapse, dopaminergic neuron differentiation, negative regulation of the fatty acid metabolic process, response to fatty acid, regulation of fatty acid transport, and regulation of fatty acid metabolic process were considered key targets, including AKT1, CASP3, VEGFA, INS, CTNNB1, PTGS2 and IL1B. PTGS2, an enzyme for prostaglandin synthesis, is involved in inflammatory and pain responses. In our study, it was involved in the pathway of the serotonergic synapse, regulation of the fatty acid metabolic process, and response to the fatty acid. Caspase-3 (CASP3) as a major activated mediator of apoptosis, was enriched in the pathway of serotonergic synapse. A previous animal study showed that weaning stress-induced diarrhea in mice, accompanied by a significant increase in 5-HT levels, caspase-3 cells, and apoptotic cells in the small intestine, thereby impairing the renewal of intestinal epithelial cells and the mucosal barrier [56]. A recent study has demonstrated that Tong-xie-yao-fang could improve IBS-D by inhibiting apoptosis including affecting by the expression of the Caspase-3 [57]. This study demonstrated the involvement of CTNNB1 and VEGFA in the process of dopaminergic neuron differentiation. Previous studies demonstrated that CTNNB1 expression was down-regulated in the colonic tissue of IBS-D patients [58]. Vascular endothelial growth factor (VEGF) is an important modulator of gastric mucosal repair, and TCM could increase the expression of VEGF to protect against gastric mucosal injury and accelerate gastric emptying [59]. AKT1, one of the key targets, belongs to AKT kinases, and AKT1 is a key protein in the PI3K/AKT signalling pathway. In lipid metabolism, Akt activation regulates a series of gene expression, including genes related to intracellular fatty acid synthesis. Inhibition of the PI3K signaling pathway could improve the symptoms by reducing the visceral hypersensitivity of IBS-D patients [60]. INS gene encodes insulin, a peptide hormone that plays a vital role in the regulation of carbohydrate and lipid metabolism. The reduction in pancreatic endocrine levels would lead to a decrease in insulin which might be associated with dyspeptic symptoms through impaired gastric emptying and duodenal inflammation [61]. Clinical research showed that decreased levels of insulin were observed in a starch- and sucrose-reduced diet intervention to IBS, and hyperinsulinemia might cause abdominal bloating, pain and diarrhea [62]. IL1B is a cytokine protein that is involved in the modulation of autoimmune inflammation. Recent studies have demonstrated that 5-HT receptor agonists and DA receptor antagonists could attenuate IL1B expression [63]. The change of IL1B expression was closely related to IBS-D, and IL1B -31C/T polymorphism played a role in chronic inflammation of the gastric mucosa in FD patients [64, 65]. The above studies confirmed that these seven key targets are involved in the regulation of 5-HT, DA, and butyrate by SLBZS and are also the therapeutic targets of SLBZS for the three diseases.

Through network analysis, we identified 14 key components, namely quercetin, kaempferol, luteolin, formononetin, diosgenin, beta-carotene, ginsenoside rf, beta-sitosterol, stigmasterol, pronuciferine, naringenin, acacetin, ginsenoside rh2, and fumarine. The molecular docking results showed that the affinities were all less than -5.8 kcal/mol, indicating a high affinity between the key components and targets. In particular, diosgenin showed the lowest average binding energy, with PTGS2-diosgenin and PTGS2-luteolin demonstrating the best binding abilities. MD simulations confirmed that the PTGS2-diosgenin and PTGS2-luteolin complexes had good binding activity, with PTGS2-diosgenin exhibiting higher structural stability and lower interaction energy. Based on this, we conducted *in vitro* validation of PTGS2-diosgenin. BLI data indicated that diosgenin can directly bind to the PTGS2 protein. Previous research showed that diosgenin downregulates COX-2 (PTGS2) *via* the glucocorticoid receptor and suppresses COX-2 in the macrophages of LPS-induced acute mouse liver injury. [66]. Our study further demonstrated that diosgenin can directly bind to PTGS2, thereby regulating its activity. Diosgenin is a natural steroidal sapogenin. Clinical studies have shown that diosgenin can improve 5-HT metabolism, exerting antidepressant effects by increasing 5-HT levels in the hippocampus [67], increasing butyrate-producing bacteria and plasma butyrate, and improving gut microbiota composition and intestinal integrity, thereby reducing fecal bacterial density and alleviating the severity of diarrhea [68, 69]. Upregulation of PTGS2 promotes the release of prostaglandin E2, resulting in increased mucosal 5-HT, which may contribute to abdominal pain in IBS-D [70]. Meanwhile, abnormal synthesis of prostaglandin E2 appears to induce visceral hypersensitivity, which may lead to abdominal pain or discomfort in FDr, IBS-D and FD [71]. Furthermore, another important component identified through molecular docking and MD simulations is luteolin. Previous research has shown that an extract containing luteolin, 36_EB, may be effective for IBS-D when gastrointestinal relaxation is needed [72]. When luteolin was combined with fiber, there were synergistic effects on the modulation of the relative abundance of different bacterial taxa. In particular, the proportion of Prevotella was significantly increased, which showed a strong correlation with gastric emptying time and the severity of IBS and FD symptoms [73-75]. These components have complex pharmacological effects, and the synergistic effects may allow for better modulation of the gut-brain axis by SLBZS.

This study employed network pharmacology, molecular docking, and molecular dynamics simulations, along with in vitro validation using BLI. However, we acknowledge that these results may not fully capture the dynamic interactions occurring in the complex biological environment of the body. Currently, the lack of appropriate animal models that mimic symptom overlap remains a challenge. Moving forward, it will be crucial to identify suitable animal models or conduct human clinical trials for *in vivo* studies to validate whether the effects observed *in vitro* on PTGS2 and other targets translate into therapeutic outcomes for FDr, IBS-D, and FD.

## CONCLUSION

In summary, based on biological information and combined with network pharmacology, computer simulation, and in vitro experiments, this study identified potential targets shared by FDr, IBS-D, and FD. The effect of diosgenin on PTGS2 may be one of the pharmacological mechanisms of SLBZS in treating these conditions, thus providing new insights into the pharmacological mechanism of SLBZS in the treatment of functional gastrointestinal disorders.

## AUTHORS’ CONTRIBUTIONS

The authors confirm their contribution to the paper as follows: study conception and design: HX; data collection: LH; draft manuscript: CM. All authors reviewed the results and approved the final version of the manuscript.

## Figures and Tables

**Fig. (1):**
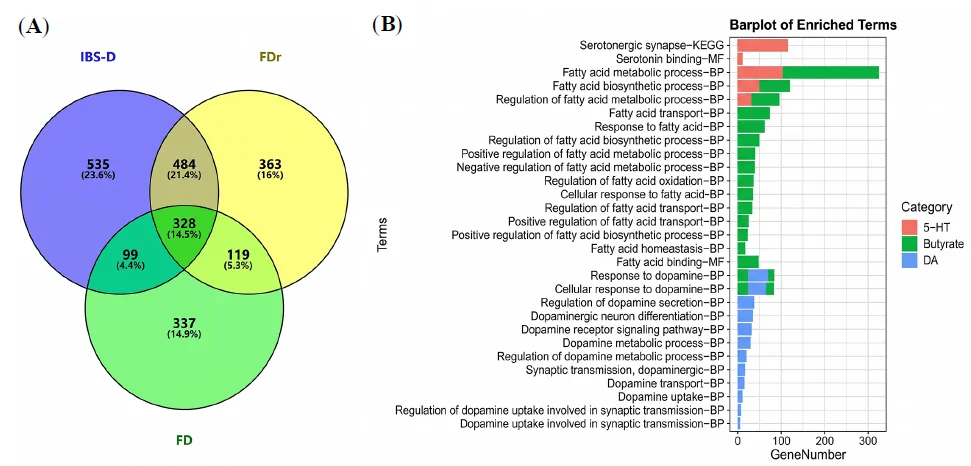
The common targets and GO and KEGG analyses for the common targets among FDr, IBS-D, and FD. (**A**) The Venn diagram. (**B**) The GO analysis and the KEGG pathway analysis enrichment in 5-HT-, DA-, butyrate-related common targets, including BP, CC, and MF.

**Fig. (2):**
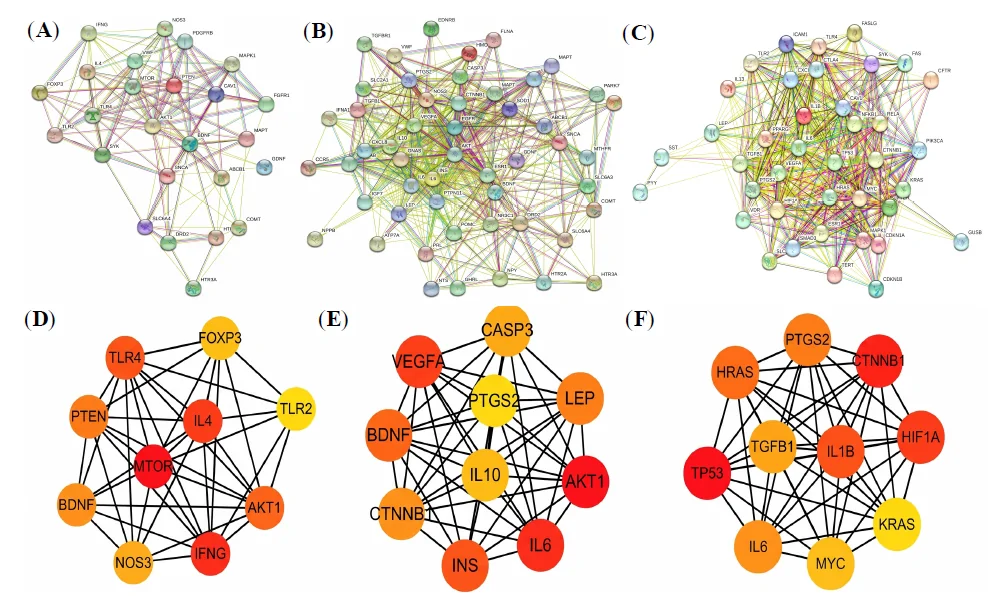
The PPI networks of 5-HT/DA/butyrate-disease targets, respectively. (**A**) The PPI network of 5-HT-diseases. (**B**) The PPI network of DA-diseases. (**C**) The PPI network of butyrate-diseases. (**D**) Hub genes of 5-HT-diseases according to MCC. (**E**) Hub genes of DA-diseases according to MCC. (**F**) Hub genes of butyrate-diseases according to MCC.

**Fig. (3):**
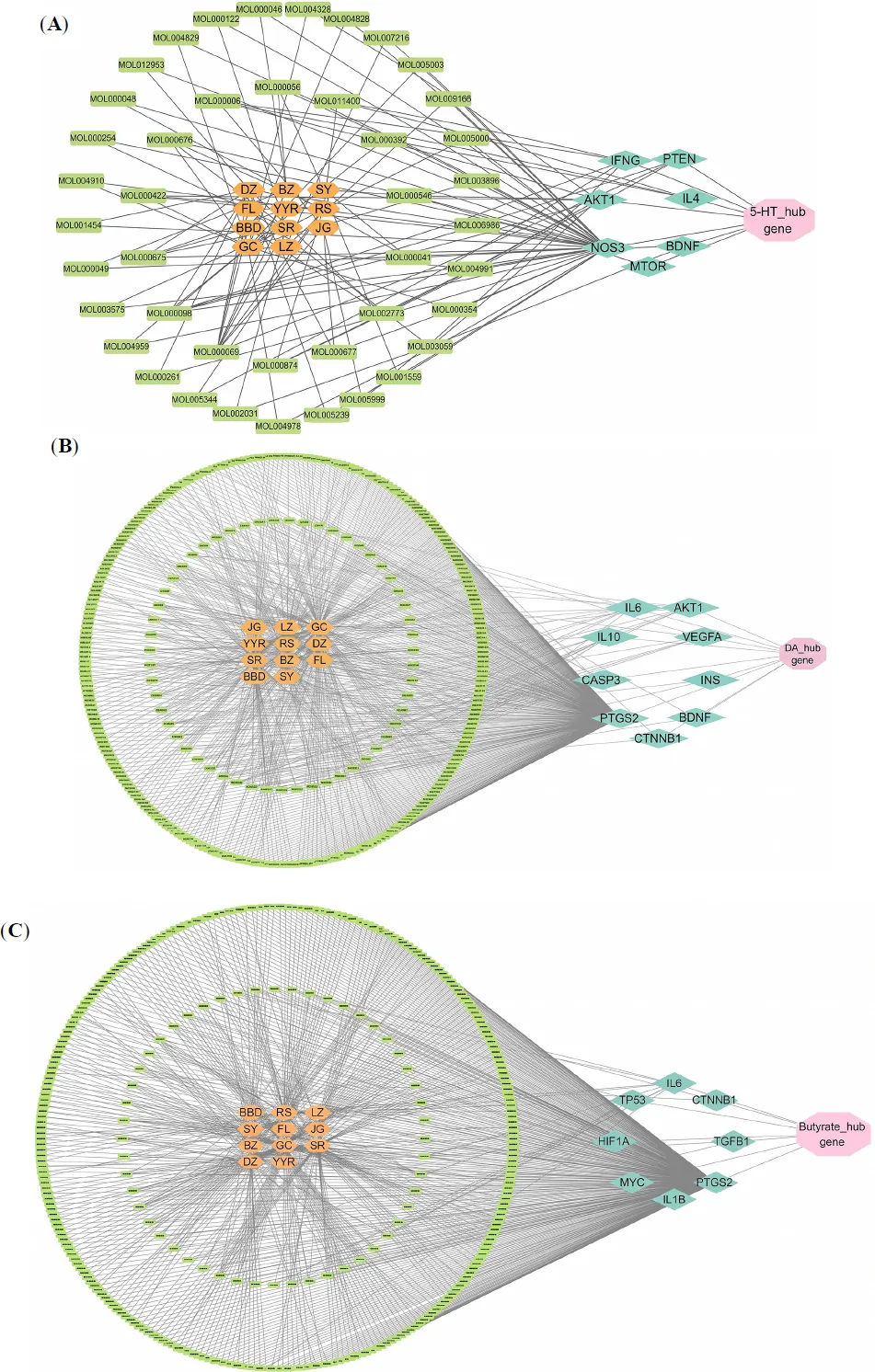
The potential therapeutic targets and the network construction. (**A**) Network of drug-ingredient-hub gene-5-HT. (**B**) Network of drug-ingredients-hub gene-DA. (**C**) Network of drug-ingredients-hub gene-butyrate.

**Fig. (4):**
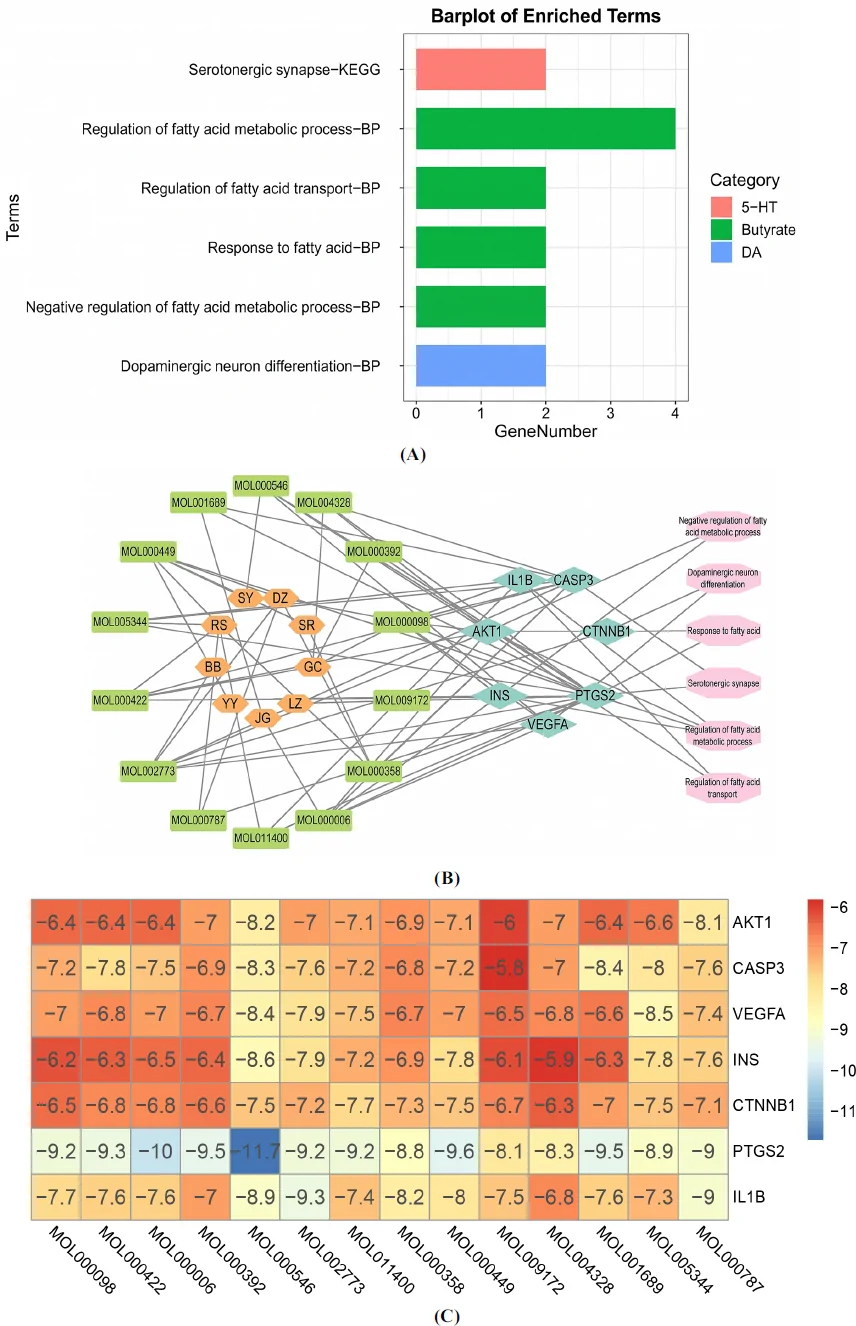
Analysis of 7 key targets and 14 key ingredients. (**A**) The GO enrichment analysis and the KEGG pathway enrichment analysis in 5-HT-, DA-, butyrate-related key targets and pathways. (**B**) Network of SLBZS-key ingredients-(H-D-B) therapeutic target-(H-D-B)- therapeutic pathway. (**C**) Heatmap of Docking affinity between 14 ingredients and 7 targets.

**Fig. (5):**
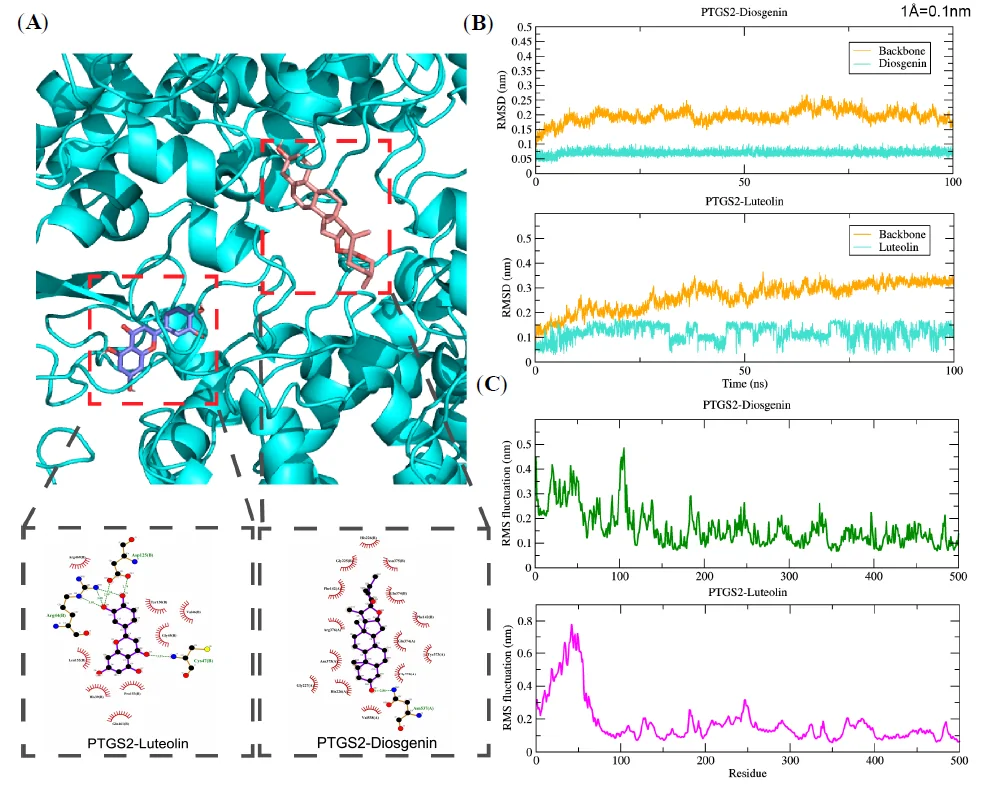
Molecular docking analysis and MD simulations. (**A**) The binding modes of PTGS2-diosgenin and PTGS2-luteolin. The purple structures represent the active ingredients, the red arcs represent amino acids that form hydrophobic interactions with the active ingredients, and the green dashed lines represent the hydrogen bonds formed between the ingredients and the targets. (**B**) RMSD of the backbones (orange) and ligands (blue) during simulations. (**C**) RMSF of the PTGS2-diosgenin (green) and PTGS2-luteolin (pink).

**Fig. (6):**
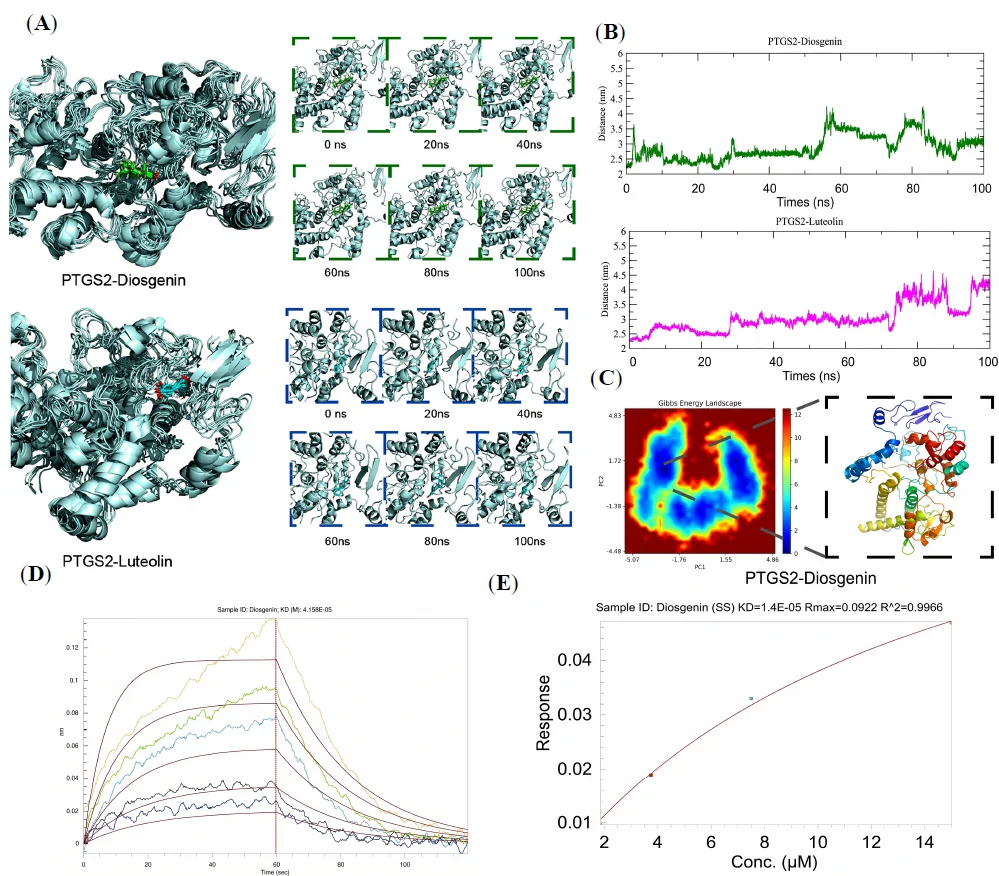
Snapshots, center-of-mass distances, and free energy landscapes. (**A**)The superimposed snapshots of the MD trajectories and binding poses at the active binding pocket of PTGS2 at different time points (0 ns, 10 ns, 20 ns, 40 ns, 60 ns, 80 ns, and 100 ns). (**B**) The center-of-mass distances of PTGS2-diosgenin (green) and PTGS2-luteolin (pink). (**C**) Principal component analysis and free energy landscapes. Blue defines the conformational space with minimum energy (stable state), while red defines the unstable state. (**D**) A dose-dependent binding of diosgenin to PTGS2. (**E**) Steady-state analysis of diosgenin and PTGS2.

**Table 1:** Intersection of the genes of 5-HT, dopamine, and butyrate with 328 common targets.

**Category**	**Gene**
5-HT-Disease	FOXP3, PTEN, AKT1, FGFR1, TLR4, IFNG, SLC6A4, ABCB1, IL4, BDNF, MAPT, HTR3A, COMT, PDGFRB, MAPK1, GDNF, TLR2, MTOR, HTR2A, SYK, CAV1, SNCA, VWF, NOS3, DRD2
DA-Disease	IL10, FLNA, IL6, TGFB1, EGFR, PTPN11, AKT1, CXCL8, ATP7A, SLC6A4, CTNNB1, UGT1A1, ABCB1, GNAS, EDNRB, BDNF, MAPT, TGFBR1, SLC2A1, IGF2, HTR3A, LEP, COMT, INS, PDGFRB, MAPK1, GDNF, MTHFR, VEGFA, GHRL, POMC, IFNA1, HTR2A, SLC6A3, LRRK2, NR3C1, NTS, PTGS2, ESR1, HMOX1, PRL, SOD1, SNCA, NPY, VWF, CCR5, NOS3, DRD2, CASP3, NPPB, PARK7
Butyrate-Disease	TP53, VEGFA, HIF1A, PTGS2, PIK3CA, IL6, TGFB1, NFKB1, ESR1, TLR4, KRAS, PPARG, VDR, IL1B, CTNNB1, PTEN, CDKN1A, CFTR, TERT, CXCL8, MYC, MAPK1, LEP, TLR2, CTLA4, ICAM1, RELA, FAS, CAV1, CDKN1B, FASLG, SLC2A1, HRAS, SMAD3, IL13, SYK, PYY, SST, GUSB

**Table 2:** Drugs and ingredients of Shen-Ling-Bai-Zhu-San.

**Category**	**Chinese Name**	**Plant Name**	**Ingredients**
Sovereign Drug	Ren Shen (RS)	*Panax ginseng C. A. Mey.*	190
	Bai Zhu (BZ)	*Atractylodes macrocephala Koidz.Macrocephalae.*	55
	Fu Ling (FL)	*Poria cocos (Schw.) Wolf.*	34
Minister Drug	Shan Yao (SY)	*Dioscorea opposita Thunb.*	71
	Lian Zi (LZ)	*Nelumbo nucifera Gaertn.*	9
	Bai Bian Dou (BBD)	*Dolichos lablab L.*	14
	Yi Yi Ren (YYR)	*Coix lacryma-jobi L.var.ma-yuen (Roman.) Stapf.*	38
Assistant Drug	Sha Ren (SR)	*Amomum villosum Lour.*	165
	Jie Geng (JG)	*Platycodon grandiflorum (Jacq.) A.DC.*	102
Envoy Drug	Gan Cao (GC)	*Glycyrrhiza uralensis Fisch.*	280
	Da Zao (DZ)	*Ziziphus jujuba Mill.*	133

**Table 3:** 7 key targets in the network.

**Name**	**Protein Names**	**PDB ID**
AKT1	RAC-alpha serine/threonine-protein kinase	1UNQ
PTGS2	Prostaglandin G/H synthase 2	5F19
CASP3	Caspase-3	1NME
VEGFA	Vascular endothelial growth factor A	1MJV
INS	Insulin	1B9E
CTNNB1	Catenin beta-1	4IGG
IL1B	Interleukin-1B	5BOW

**Table 4:** 14 key ingredients in the network.

**Mol ID**	**Molecule Name**	**OB (%)**	**DL**	**Herb**
MOL000098	Quercetin	46.43	0.28	GC DZ
MOL000422	Kaempferol	41.88	0.24	RS GC
MOL000006	Luteolin	36.16	0.25	JG
MOL000392	Formononetin	69.67	0.21	GC
MOL000546	Diosgenin	80.88	0.81	SY
MOL002773	Beta-carotene	37.18	0.58	BBD DZ
MOL011400	Ginsenoside rf	17.74	0.24	RS
MOL000358	Beta-sitosterol	36.91	0.75	RS SR DZ
MOL000449	Stigmasterol	43.83	0.76	RS SY YYR DZ
MOL009172	Pronuciferine	32.75	0.37	LZ
MOL004328	Naringenin	59.29	0.21	GC
MOL001689	Acacetin	34.97	0.24	JG
MOL005344	Ginsenoside rh2	36.32	0.56	RS
MOL000787	Fumarine	59.26	0.83	RS DZ

## Data Availability

All data generated or analyzed during this study are included in this published article and its supplementary information files.

## LIST OF ABBREVIATIONS

5-HT: 5-Hydroxytryptamine
BBD: BaiBianDou
BLI: Bio-Layer Interferometry
BP: Biological Processes
BZ: BaiZhu
CC: Cellular Components
DA: Dopamine
DGBIs: Disorders of Gut-Brain Interaction
DL: Drug-Likeness
DZ: DaZao
ETCM: The Encyclopedia of Traditional Chinese Medicine
FD: Functional Dyspepsia
FDr: Functional Diarrhea
FGIDs: Functional gastrointestinal diseases
FL: FuLing
GC: GanCao
GO: Gene Ontology
IBS-D: Irritable Bowel Syndrome with Predominant Diarrhea
JG: JieGeng
KEGG: Kyoto Encyclopedia of Genes and Genome
LZ: LianZi
MD: Molecular Dynamic
MF: Molecular Functions
OB: Oral Bioavailability
PPI: Protein-Protein Interaction
RS: RenShen
SCFAs: Short-Chain Fatty Acids
SLBZS: Shen-Ling-Bai-Zhu-San
SR: ShaRen
SSRD: Starch- and Sucrose-Reduced Diet
SY: ShanYao
TCM: Traditional Chinese Medicine
TCMSP: Traditional Chinese Medicine Systems Pharmacology Database and Analysis Platform
Uniprot: Universal Protein Resource Knowledge base
YYR: YiYiRen

## SUPPLEMENTARY MATERIAL

Supplementray material is available on the publisher's website along with the published article.
