# Supplementary material for: Exploring the Comorbidity Mechanism among Functional Diarrhea, Irritable Bowel Syndrome with Predominant Diarrhea, and Functional Dyspepsia through the Gut-brain Axis and the Pharmacological Mechanisms of Shen-Ling-Bai-Zhu-San Therapy
Source: Comb Chem High Throughput Screen. 2025 Apr 18;29(16):2273–88. doi: 10.2174/0113862073329698250102120037 (PMC13684977; doi:10.2174/0113862073329698250102120037)
Supplement: Supplementary file 1 [file CCHTS-29-16-2273_SD1.pdf]

Supplementary Material

Exploring the Comorbidity Mechanism among Functional Diarrhea, Irritable Bowel Syndrome with Predominant Diarrhea, and Functional Dyspepsia through the Gut-brain Axis and the Pharmacological Mechanisms of Shen-Ling-Bai-Zhu-San Therapy

Chun-feng Mei[1], Li-li Han[1] and Hong Xue[1,*]

[1]*Digestive Laboratory of Traditional Chinese Medicine Research Institute of Spleen and Stomach Diseases, Xi-yuan Hospital, China Academy of Chinese Medical Sciences, Beijing, 100091, China*

S1 197 BP terms, 193 CC terms, 305 MF terms, and 207 KEGG enrichment pathways.

Term	Description
BP	Pathways in cancer, JAK-STAT signaling pathway, PI3K-Akt signaling pathway, Proteoglycans in cancer, Gastric cancer, EGFR tyrosine kinase inhibitor resistance, Prostate cancer, Pancreatic cancer, MAPK signaling pathway, FoxO signaling pathway, Colorectal cancer, Melanoma, Chronic myeloid leukemia, Endocrine resistance, Bladder cancer, Breast cancer, Central carbon metabolism in cancer, Hepatocellular carcinoma, Endometrial cancer, Ras signaling pathway, Rap1 signaling pathway, Non-small cell lung cancer, Glioma, Chemical carcinogenesis - receptor activation, Thyroid hormone signaling pathway, Acute myeloid leukemia, Renal cell carcinoma, Focal adhesion, Prolactin signaling pathway, Relaxin signaling pathway, Phospholipase D signaling pathway, ErbB signaling pathway, Chemical carcinogenesis - reactive oxygen species, Signaling pathways regulating pluripotency of stem cells, Chemokine signaling pathway, Thyroid cancer, Fc epsilon RI signaling pathway, Growth hormone synthesis, secretion and action, Autophagy - animal, mTOR signaling pathway, Choline metabolism in cancer, Regulation of actin cytoskeleton, VEGF signaling pathway, B cell receptor signaling pathway, Estrogen signaling pathway, Apelin signaling pathway, Melanogenesis, Gap junction, Insulin signaling pathway, Axon guidance, GnRH secretion, Serotonergic synapse, Long-term depression, Oxytocin signaling pathway, Cholinergic synapse, GnRH signaling pathway, Alcoholism, Long-term potentiation, AGE-RAGE signaling pathway in diabetic complications, Human T-cell leukemia virus 1 infection, Human cytomegalovirus infection, Hepatitis B, Kaposi sarcoma-associated herpesvirus infection, Human papillomavirus infection, Lipid and atherosclerosis, Cellular senescence, Influenza A, Chagas disease, PD-L1 expression and PD-1 checkpoint pathway in cancer, Epstein-Barr virus infection, T cell receptor signaling pathway, Hepatitis C, Measles, Human immunodeficiency virus 1 infection, C-type lectin receptor signaling pathway, Viral carcinogenesis, Apoptosis, Fluid shear stress and atherosclerosis, Natural killer cell mediated cytotoxicity, Coronavirus disease - COVID-19, Osteoclast differentiation, Neurotrophin signaling pathway, Yersinia infection, Herpes simplex virus 1 infection, Shigellosis, Salmonella infection, Amoebiasis, Sphingolipid signaling pathway, HIF-1 signaling pathway, MicroRNAs in cancer, Tuberculosis, Inflammatory bowel disease, Th17 cell differentiation, Toxoplasmosis, Leishmaniasis, Th1 and Th2 cell differentiation, Cytokine-cytokine receptor interaction, Viral protein interaction with cytokine and cytokine receptor, Platinum drug resistance, Small cell lung cancer, p53 signaling pathway, TNF signaling pathway, NF-kappa B signaling pathway, Toll-like receptor signaling pathway, IL-17 signaling pathway, Pathogenic Escherichia coli infection, NOD-like receptor signaling pathway, Alcoholic liver disease, Legionellosis, Pertussis, Antifolate resistance, Cytosolic DNA-sensing pathway, RIG-I-like receptor signaling pathway, Malaria, Rheumatoid arthritis, African trypanosomiasis, Pathways of neurodegeneration - multiple diseases, Non-alcoholic fatty liver disease, Alzheimer disease, Prion disease, Amyotrophic lateral sclerosis, Parkinson disease, Huntington disease, Allograft rejection, Autoimmune thyroid disease, Graft-versus-host disease, Type I diabetes mellitus, Viral myocarditis, Intestinal immune network for IgA production, Cell adhesion molecules, Asthma, Antigen processing and presentation, Phagosome, Systemic lupus erythematosus, Staphylococcus aureus infection, cAMP signaling pathway, Neuroactive ligand-receptor interaction, Hematopoietic cell lineage, Adipocytokine signaling pathway, Insulin resistance, Diabetic cardiomyopathy, Longevity regulating pathway, AMPK signaling pathway, Neutrophil extracellular trap formation, Longevity regulating pathway - multiple species, Type II diabetes mellitus, Platelet activation, Regulation of lipolysis in adipocytes, Fc gamma R-mediated phagocytosis, Aldosterone-regulated sodium reabsorption, Inflammatory mediator regulation of TRP channels, Progesterone-mediated oocyte maturation, Carbohydrate digestion and absorption, Adherens junction, Cell cycle, TGF-beta signaling pathway, Hippo signaling pathway, Wnt signaling pathway, Necroptosis, Apoptosis - multiple species, Transcriptional misregulation in cancer, Calcium signaling pathway, Cushing syndrome, Parathyroid hormone synthesis, secretion and action, Primary immunodeficiency, Homologous recombination, Fanconi anemia pathway, Bile secretion, ABC transporters, Vascular smooth muscle contraction, cGMP-PKG signaling pathway, Epithelial cell signaling in Helicobacter pylori infection, Endocytosis, Thermogenesis, Basal cell carcinoma, Hedgehog signaling pathway, Complement and coagulation cascades, Leukocyte transendothelial migration, Bacterial invasion of epithelial cells, Phosphatidylinositol signaling system, Inositol phosphate metabolism, Mitophagy - animal, Mismatch repair, Ferroptosis, Mineral absorption, Nucleo-

	tide excision repair, Base excision repair, Cocaine addiction, Hypertrophic cardiomyopathy, Dilated cardiomyopathy, Tight junction, Renin secretion, Ovarian steroidogenesis, Cholesterol metabolism, Fat digestion and absorption, PPAR signaling pathway, Viral life cycle - HIV-1, Notch signaling pathway, Glucagon signaling pathway, Insulin secretion, Porphyrin metabolism, Drug metabolism - other enzymes, Steroid hormone biosynthesis, Arginine biosynthesis, ECM-receptor interaction, Pancreatic secretion, Gastric acid secretion
CC	membrane raft, membrane microdomain, caveola, plasma membrane raft, side of membrane, external side of plasma membrane, receptor complex, plasma membrane protein complex, plasma membrane signaling receptor complex, endocytic vesicle, endocytic vesicle membrane, coated vesicle, clathrin-coated endocytic vesicle, clathrin-coated endocytic vesicle membrane, clathrin-coated vesicle, early endosome, coated vesicle membrane, clathrin-coated vesicle membrane, endosome membrane, vesicle lumen, cytoplasmic vesicle lumen, secretory granule lumen, platelet alpha granule, platelet alpha granule lumen, perinuclear region of cytoplasm, cell body, axon, neuronal cell body, dendrite, dendritic tree, apical part of cell, apical plasma membrane, basal part of cell, basal plasma membrane, basolateral plasma membrane, endoplasmic reticulum lumen, extracellular matrix, external encapsulating structure, collagen-containing extracellular matrix, endocytic vesicle lumen, chylomicron, very-low-density lipoprotein particle, triglyceride-rich plasma lipoprotein particle, high-density lipoprotein particle, endoplasmic reticulum exit site, plasma lipoprotein particle, lipoprotein particle, protein-lipid complex, blood microparticle, lytic vacuole, lysosome, vacuolar lumen, azurophil granule lumen, primary lysosome, azurophil granule, PML body, chromosome, telomeric region, chromosomal region, BRCA1-C complex, replication fork, site of DNA damage, site of double-strand break, phagocytic vesicle, cell-cell junction, lateral plasma membrane, flotillin complex, apical junction complex, Wnt signalosome, bicellular tight junction, tight junction, adherens junction, catenin complex, cell leading edge, actin-based cell projection, lamellipodium, filopodium, actin cytoskeleton, transcription regulator complex, transcription repressor complex, RNA polymerase II transcription regulator complex, transport vesicle, MHC protein complex, Golgi membrane, integral component of lumenal side of endoplasmic reticulum membrane, lumenal side of endoplasmic reticulum membrane, lumenal side of membrane, MHC class II protein complex, ER to Golgi transport vesicle membrane, MHC class I peptide loading complex, COPII-coated ER to Golgi transport vesicle, MHC class I protein complex, transport vesicle membrane, phagocytic vesicle membrane, integral component of endoplasmic reticulum membrane, intrinsic component of endoplasmic reticulum membrane, early endosome membrane, cell-substrate junction, focal adhesion, distal axon, neuron projection terminus, axon terminus, presynapse, terminal bouton, endosome lumen, postsynapse, glutamatergic synapse, extrinsic component of membrane, cytoplasmic side of membrane, extrinsic component of plasma membrane, cytoplasmic side of plasma membrane, extrinsic component of cytoplasmic side of plasma membrane, HFE-transferrin receptor complex, recycling endosome, recycling endosome membrane, death-inducing signaling complex, inclusion body, aggresome, transferase complex, transferring phosphorus-containing groups, protein kinase complex, serine/threonine protein kinase complex, mismatch repair complex, cell projection membrane, leading edge membrane, germ cell nucleus, lateral element, synaptonemal complex, synaptonemal structure, DNA repair complex, nuclear ubiquitin ligase complex, condensed nuclear chromosome, male germ cell nucleus, condensed chromosome, nuclear chromosome, immunological synapse, organelle outer membrane, outer membrane, mitochondrial outer membrane, mitochondrial envelope, mitochondrial membrane, intercellular canaliculus, cell cortex, sarcomere, myofibril, contractile fiber, cortical cytoskeleton, secretory granule membrane, platelet alpha granule membrane, microvillus, late endosome, cytochrome complex, mitochondrial respiratory chain complex III, respiratory chain complex III, respiratory chain complex, mitochondrial respirasome, respirasome, phosphatidylinositol 3-kinase complex, class IA, phosphatidylinositol 3-kinase complex, class I, phosphatidylinositol 3-kinase complex, nucleotide-excision repair complex, perikaryon, growth cone, site of polarized growth, axonal growth cone, tertiary granule lumen, tertiary granule, specific granule lumen, specific granule, nuclear envelope, nuclear membrane, serine-type peptidase complex, apicolateral plasma membrane, euchromatin, female germ cell nucleus, brush border, cluster of actin-based cell projections, brush border membrane, spanning component of plasma membrane, spanning component of membrane, ficolin-1-rich granule, ficolin-1-rich granule lumen, Golgi lumen, glycoprotein complex, intrinsic component of external side of plasma membrane, intercalated disc, cell-cell contact zone, trans-Golgi network, dense core granule, podosome, lysosomal lumen, presynaptic membrane, integral component of presynaptic membrane, spindle, Schaffer collateral - CA1 synapse
MF	signaling receptor activator activity， receptor ligand activity， signaling receptor regulator activity， cytokine receptor binding， cytokine activity， growth factor activity， growth factor receptor binding， kinase binding， protein kinase binding， hormone activity， G protein-coupled receptor binding， neuropeptide receptor binding， neuropeptide hormone activity， protein kinase activity， phosphotransferase activity, alcohol group as acceptor， kinase activity， protein serine/threonine kinase activity， protein serine kinase activity， protease binding， protein homodimerization activity， transcription factor binding， chromatin binding， DNA-binding transcription factor binding ， RNA polymerase II-specific DNA-binding transcription factor binding， transcription coregulator binding， transcription coactivator binding， nuclear receptor binding， DNA-binding transcription activator activity, RNA polymerase II-specific， DNA-binding transcription activator activity， protein C-terminus binding， protein domain specific binding， chaperone binding， cytokine binding， immune receptor activity， cytokine receptor activity， exogenous protein binding， virus receptor activity， coreceptor activity， chemokine binding， C-C chemokine receptor activity， C-C chemokine binding， G protein-coupled chemoattractant receptor activity， chemokine receptor activity， kinase regulator activity， protein kinase regulator activity， kinase activator activity， enzyme activator activity， protein kinase activator activity， protein tyrosine kinase activator activity， protein tyrosine kinase activity， growth factor binding， transmembrane receptor protein kinase activity， transmembrane receptor protein tyrosine kinase activity， fibroblast growth factor receptor activity， fibroblast growth factor binding， glycosaminoglycan binding， heparin binding， sulfur compound binding， protein N-terminus binding， ubiquitin-like protein ligase binding， ubiquitin protein ligase binding， phosphatase binding， protein phosphatase binding， protein phosphatase 2A binding， damaged DNA binding， catalytic activity, acting on DNA， ATP-dependent activity, acting on DNA， ATP-dependent activity， catalytic activity, acting on a nucleic acid， ATP hydrolysis activity， DNA helicase activity， ribonucleoside triphosphate phosphatase activity， pyrophosphatase activity， hydrolase activity, acting on acid anhydrides， hydrolase activity, acting on acid anhydrides, in phosphorus-containing anhydrides， helicase activity， cell adhesion molecule binding， integrin

	binding, cadherin binding, insulin-like growth factor receptor binding, insulin receptor binding, peptide binding, amide binding, amyloid-beta binding, copper ion binding, cuprous ion binding, metallochaperone activity, steroid binding, patched binding, cholesterol binding, sterol binding, hormone receptor binding, peptide hormone receptor binding, tumor necrosis factor receptor superfamily binding, death receptor binding, tumor necrosis factor receptor binding, p53 binding, molecular function activator activity, scaffold protein binding, MAP kinase kinase activity, protein serine/threonine/tyrosine kinase activity, mismatch repair complex binding, mismatched DNA binding, single-stranded DNA binding, guanine/thymine mispair binding, oxidized purine DNA binding, ATP-dependent DNA damage sensor activity, oxidized DNA binding, small molecule sensor activity, single base insertion or deletion binding, DNA damage sensor activity, DNA insertion or deletion binding, four-way junction DNA binding, DNA secondary structure binding, MutSalpha complex binding, ADP binding, nuclear receptor activity, ligand-activated transcription factor activity, carboxylic acid binding, organic acid binding, monocarboxylic acid binding, nuclear retinoid X receptor binding, fatty acid binding, nuclear retinoic acid receptor binding, general transcription initiation factor binding, promoter-specific chromatin binding, RNA polymerase II general transcription initiation factor binding, single-stranded DNA endodeoxyribonuclease activity, TFIID-class transcription factor complex binding, deoxyribonuclease activity, basal transcription machinery binding, basal RNA polymerase II transcription machinery binding, endodeoxyribonuclease activity, cyclin-dependent protein serine/threonine kinase inhibitor activity, cyclin-dependent protein serine/threonine kinase regulator activity, protein serine/threonine kinase inhibitor activity, protein kinase inhibitor activity, kinase inhibitor activity, molecular function inhibitor activity, cyclin binding, heat shock protein binding, Hsp90 protein binding, Hsp70 protein binding, insulin receptor substrate binding, hormone binding, phosphatidylinositol 3-kinase binding, peptide hormone binding, phospholipase activator activity, SH2 domain binding, lipase activator activity, non-membrane spanning protein tyrosine kinase activity, phospholipase binding, antioxidant activity, oxygen binding, peroxidase activity, oxidoreductase activity, acting on peroxide as acceptor, heme binding, tetrapyrrole binding, oxidoreductase activity, monooxygenase activity, oxidoreductase activity, acting on paired donors, with incorporation or reduction of molecular oxygen, NAD(P)H as one donor, and incorporation of one atom of oxygen, beta-catenin binding, phosphoprotein binding, phosphotyrosine residue binding, protein tyrosine kinase binding, protein phosphorylated amino acid binding, receptor tyrosine kinase binding, transcription coregulator activity, transcription coactivator activity, transcription corepressor activity, protein self-association, enzyme inhibitor activity, endopeptidase inhibitor activity, peptidase inhibitor activity, peptidase regulator activity, endopeptidase regulator activity, chemoattractant activity, MHC protein complex binding, MHC class II protein complex binding, peptide antigen binding, antigen binding, MHC class II receptor activity, T cell receptor binding, 1-phosphatidylinositol-3-kinase activity, phosphatidylinositol 3-kinase activity, 1-phosphatidylinositol-4-phosphate 3-kinase activity, phosphatidylinositol-4,5-bisphosphate 3-kinase activity, phosphatidylinositol kinase activity, phosphatidylinositol-3,4-bisphosphate 5-kinase activity, phosphatidylinositol bisphosphate kinase activity, phosphatidylinositol phosphate kinase activity, transforming growth factor beta receptor binding, type II transforming growth factor beta receptor binding, transforming growth factor beta binding, type I transforming growth factor beta receptor binding, transforming growth factor beta receptor activity, activin binding , transmembrane receptor protein serine/threonine kinase activity, Toll-like receptor binding, lipopolysaccharide binding, pattern recognition receptor activity, hydrolase activity, hydrolyzing N-glycosyl compounds, molecular adaptor activity, protein-macromolecule adaptor activity, signaling adaptor activity, amine binding, serotonin binding, organic hydroxy compound transmembrane transporter activity, solute:sodium symporter activity, MAP kinase kinase kinase activity, lipid transporter activity, xenobiotic transmembrane transporter activity, phosphatidylcholine floppase activity, ABC-type transporter activity, ABC-type xenobiotic transporter activity, glycerophospholipid flippase activity, floppase activity, ATPase-coupled transmembrane transporter activity, phosphatidylcholine transporter activity, flippase activity, ATPase-coupled intramembrane lipid transporter activity, ErbB-3 class receptor binding, protein heterodimerization activity, I-SMAD binding, SMAD binding, R-SMAD binding, collagen binding, lipoprotein particle binding, protein-lipid complex binding, lipoprotein particle receptor binding, cholesterol transfer activity, sterol transfer activity, low-density lipoprotein particle receptor binding, phospholipid binding, sterol transporter activity, primary active transmembrane transporter activity, active transmembrane transporter activity, active ion transmembrane transporter activity, oxidoreduction-driven active transmembrane transporter activity, NF-kappaB binding, nitric-oxide synthase regulator activity, ATPase binding, nuclear estrogen receptor binding, chromatin DNA binding, bHLH transcription factor binding, histone acetyltransferase activity, peptide-lysine-N-acetyltransferase activity, mRNA 3'-UTR binding, mRNA base-pairing post-transcriptional repressor activity , translation repressor activity, mRNA binding, 4 iron, 4 sulfur cluster binding, 3'-5'-exodeoxyribonuclease activity, iron-sulfur cluster binding, metal cluster binding, exodeoxyribonuclease activity, exodeoxyribonuclease activity, producing 5'-phosphomonoesters, DNA polymerase activity, histone acetyltransferase binding, core promoter sequence-specific DNA binding, histone deacetylase binding, DNA-binding transcription repressor activity, calcium ion binding, glycosphingolipid binding, carbohydrate binding, sphingolipid binding, glycolipid binding, tumor necrosis factor receptor activity, death receptor activity, platelet-derived growth factor binding, platelet-derived growth factor receptor binding, death domain binding, neurotrophin receptor binding, serine hydrolase activity, endopeptidase activity, serine-type peptidase activity, peptidase activity, serine-type endopeptidase activity, nitric-oxide synthase binding, pre-mRNA intronic binding, molecular condensate scaffold activity, transmembrane transporter binding, protein kinase C binding, interleukin-1 receptor binding, G protein activity, glutamate receptor binding, ionotropic glutamate receptor binding, actin binding, tau protein binding, hydrolase activity, acting on ester bonds, phosphatidylinositol phospholipase C activity, phospholipase C activity, phospholipase activity, phosphoric ester hydrolase activity, protein serine/threonine kinase activator activity, nuclear androgen receptor binding, extracellular matrix binding, magnesium ion binding, ubiquitin binding, ubiquitin-like protein binding, channel regulator activity, disordered domain specific binding, iron ion binding, actinin binding, chemokine receptor binding
KEGG	Pathways in cancer, JAK-STAT signaling pathway, PI3K-Akt signaling pathway, Proteoglycans in cancer, Gastric cancer, EGFR tyrosine kinase inhibitor resistance, Prostate cancer, Pancreatic cancer, MAPK signaling pathway, FoxO signaling pathway, Colorectal cancer, Melanoma, Chronic myeloid leukemia, Endocrine resistance, Bladder cancer, Breast cancer, Central carbon

	metabolism in cancer，Hepatocellular carcinoma，Endometrial cancer，Ras signaling pathway，Rap1 signaling pathway，Non-small cell lung cancer，Glioma，Chemical carcinogenesis - receptor activation，Thyroid hormone signaling pathway，Acute myeloid leukemia，Renal cell carcinoma，Focal adhesion，Prolactin signaling pathway，Relaxin signaling pathway，Phospholipase D signaling pathway，ErbB signaling pathway，Chemical carcinogenesis - reactive oxygen species，Signaling pathways regulating pluripotency of stem cells，Chemokine signaling pathway，Thyroid cancer，Fc epsilon RI signaling pathway，Growth hormone synthesis, secretion and action，Autophagy - animal，mTOR signaling pathway，Choline metabolism in cancer，Regulation of actin cytoskeleton，VEGF signaling pathway，B cell receptor signaling pathway，Estrogen signaling pathway，Apelin signaling pathway，Melanogenesis，Gap junction，Insulin signaling pathway，Axon guidance，GnRH secretion，Serotonergic synapse，Long-term depression，Oxytocin signaling pathway，Cholinergic synapse，GnRH signaling pathway，Alcoholism，Long-term potentiation，AGE-RAGE signaling pathway in diabetic complications，Human T-cell leukemia virus 1 infection，Human cytomegalovirus infection，Hepatitis B，Kaposi sarcoma-associated herpesvirus infection，Human papillomavirus infection，Lipid and atherosclerosis，Cellular senescence，Influenza A，Chagas disease，PD-L1 expression and PD-1 checkpoint pathway in cancer，Epstein-Barr virus infection，T cell receptor signaling pathway，Hepatitis C，Measles，Human immunodeficiency virus 1 infection，C-type lectin receptor signaling pathway，Viral carcinogenesis，Apoptosis，Fluid shear stress and atherosclerosis，Natural killer cell mediated cytotoxicity，Coronavirus disease - COVID-19，Osteoclast differentiation，Neurotrophin signaling pathway，Yersinia infection，Herpes simplex virus 1 infection，Shigellosis，Salmonella infection，Amoebiasis，Sphingolipid signaling pathway，HIF-1 signaling pathway，MicroRNAs in cancer，Tuberculosis，Inflammatory bowel disease，Th17 cell differentiation，Toxoplasmosis，Leishmaniasis，Th1 and Th2 cell differentiation，Cytokine-cytokine receptor interaction，Viral protein interaction with cytokine and cytokine receptor，Platinum drug resistance，Small cell lung cancer，p53 signaling pathway，TNF signaling pathway，NF-kappa B signaling pathway，Toll-like receptor signaling pathway，IL-17 signaling pathway，Pathogenic Escherichia coli infection，NOD-like receptor signaling pathway，Alcoholic liver disease，Legionellosis，Pertussis，Antifolate resistance，Cytosolic DNA-sensing pathway，RIG-I-like receptor signaling pathway，Malaria，Rheumatoid arthritis，African trypanosomiasis，Pathways of neurodegeneration - multiple diseases，Non-alcoholic fatty liver disease，Alzheimer disease，Prion disease，Amyotrophic lateral sclerosis，Parkinson disease，Huntington disease，Allograft rejection，Autoimmune thyroid disease，Graft-versus-host disease，Type I diabetes mellitus，Viral myocarditis，Intestinal immune network for IgA production，Cell adhesion molecules，Asthma，Antigen processing and presentation，Phagosome，Systemic lupus erythematosus，Staphylococcus aureus infection，cAMP signaling pathway，Neuroactive ligand-receptor interaction，Hematopoietic cell lineage，Adipocytokine signaling pathway，Insulin resistance，Diabetic cardiomyopathy，Longevity regulating pathway，AMPK signaling pathway，Neutrophil extracellular trap formation，Longevity regulating pathway - multiple species，Type II diabetes mellitus，Platelet activation，Regulation of lipolysis in adipocytes，Fc gamma R-mediated phagocytosis，Aldosterone-regulated sodium reabsorption，Inflammatory mediator regulation of TRP channels，Progesterone-mediated oocyte maturation，Carbohydrate digestion and absorption，Adherens junction，Cell cycle，TGF-beta signaling pathway，Hippo signaling pathway，Wnt signaling pathway，Necroptosis，Apoptosis - multiple species，Transcriptional misregulation in cancer，Calcium signaling pathway，Cushing syndrome，Parathyroid hormone synthesis, secretion and action，Primary immunodeficiency，Homologous recombination，Fanconi anemia pathway，Bile secretion，ABC transporters，Vascular smooth muscle contraction，cGMP-PKG signaling pathway，Epithelial cell signaling in Helicobacter pylori infection，Endocytosis，Thermogenesis，Basal cell carcinoma，Hedgehog signaling pathway，Complement and coagulation cascades，Leukocyte transendothelial migration，Bacterial invasion of epithelial cells，Phosphatidylinositol signaling system，Inositol phosphate metabolism，Mitophagy - animal，Mismatch repair，Ferroptosis，Mineral absorption，Nucleotide excision repair，Base excision repair，Cocaine addiction，Hypertrophic cardiomyopathy，Dilated cardiomyopathy，Tight junction，Renin secretion，Ovarian steroidogenesis，Cholesterol metabolism，Fat digestion and absorption，PPAR signaling pathway，Viral life cycle - HIV-1，Notch signaling pathway，Glucagon signaling pathway，Insulin secretion，Porphyrin metabolism，Drug metabolism - other enzymes，Steroid hormone biosynthesis，Arginine biosynthesis，ECM-receptor interaction，Pancreatic secretion，Gastric acid secretion

S2 616 ingredient–related targets.

Targets
PTGS1, PTGS2, SLC6A2, RHO, LGHG1, NCOA2, PTPN1, HSP90AB1, CALM, GABRA2, GABRA1, RXRA, ABAT, GOT1, SRC, BCHE, ADH1C, AGXT, TPI1, ME2, ME3, ADSS2, MAP1B, CHRM1, CA2, MAOB, MAOA, CHRM2, GABRA6, GABRA5, GABRA3, GRIA2, AR, F7, TOP2, PRSS1, CHRM3, ADRA1A, ADRA1B, SLC6A3, ADRB2, SLC6A4, ADRB1, ADRA2A, ADRA2C, HTR2A, ADRA2B, SCN5A, LTA4H, HTR1A, PDE3A, PGR, NR3C2, NCOA1, AKR1B1, PLAU, CTRB1, CHRNA7, PIK3CG, KCNH2, DRD1, CHRM4, CHRNA2, OPRM1, BCL2, BAX, CASP9, FOS, CASP3, CASP8, PRKCA, TGFB1, PON1, MAP2, IL6, HTR3A, ADRA1D, DPP4, MAPK14, PKIA, NOS2, PPARG, NOS3, ACHE, RELA, IKBKB, AKT1, TNF, AHSA1, MAPK8, XDH, MMP1, STAT1, CDK1, NR1I3, INSR, DIO1, PPP3CA, GSTM1, GSTM2, AKR1C3, SLPI, PLA2G1B, GLRA1, PRSS3, LCT, RXRG, CYP3A4, IL1B, IFNG, IL4, NFKBIA, CASP1, ADCYAP1, PSMG1, MAP2K4, SLC2A4, NOS1, PYGM, CTSD, GLB1, IGHG2, MAN2A1, ACP3, NR3C1, MMP9, ADH1A, KCNMA1, CDKN1A, EIF6, RB1, TP53, TEP1, RUNX1T1, CCNB1, RHOA, GSK3B, IL10, COL1A1, PTEN, GUSBP1, SLC22A5, PCYT1A, CHRM5, OPRD1, CACNA1S, KDR, MAPK1, ICAM1, IL2, TYR, PTGER3, REN, CA1, ERVK-9, ALDH5A1, KYNU, MMP8, MMP12, ARG1, CAT, RRM1, TKFC, F13A1, GOT2, GPT, CTH, AGXT2, ME1, AARS1, GATM, NFS1, SLC36A1, LDHA, GPI, LDHB, OAT, GALE, CDC25B, CELA1, GCAT, VEGFA, PGF, ALDH2, SHMT2, SLC6A9, LTF, IKKbeta, AMY2A, DHODH, PFAS, HAO1, MARS1, ACAT2, SDHA, MTR, SHMT1, GLUD1, PYGL, ASNS, CPT2, GARS1, GNMT, GLYAT, NAGS, GPT2, GLYATL1, GPHN, PLA2G2E, PIPOX, SLC25A10, YARS2, GLUD2, ATP2C1, ADSS1, GLRA2, PDHB, ALAS2, GLRA3, ALAS1, GRIA1, OPLAH, SLC25A13, PC, GRIK2, ARG2, OTC, MAT1A, SLC7A1, SLC7A4, IFNAR2, PLA2G2A, PPIA, DAO, PPIF, P4HA1, SDS, CKM, ALOX5, P3H3, CAD, SLC25A12, MAT2A, BCAT1,

DTYMK, DHCR7, CXCL8, PPARA, EP300, FABP1, RBP2, DUOX2, CD80, CD86, CD40, SAA1, VCP, SLC6A1, AMD1, SARS1, MAN1B1, ESR1, ESR2, AMY1A, FASN, SOD1, HIF1A, NR1I2, PLA2G4A, MTOR, PIM1, CCNA2, HTR2C, DRD3, HTR1B, DRD5, DRD2, COMT, MYB, TH, DDC, TRPV1, LCAT, CHEK1, ISYNA1, PCNA, MYC, HNF4A, UCP2, HNF1A, ACTB, TRIM26, F3, MAPK10, ABI1, CD36, EDN1, ERBB2, LPL, GAP43, SERPINE1, BDNF, HMGCR, MPO, PPARD, CRP, INS, PLG, ENPEP, SOAT1, CCK, CITED1, NTRK2, PDX1, SLC2A2, PAM, SCD, UCP3, CETP, PYY, DNPEP, SP1, ENPP7, RXRB, MMP2, HMOX1, CYP1A2, CAV1, CTNNB1, CASP7, GJA1, MMP10, APOB, ATP5F1B, HP, PDHX, MOGAT2, PPARGC1A, SLC12A2, GLA, TYRP1, CCND1, MAPK3, CDK4, BAK1, PRKCB, CYP2B6, SI, ARF1, GSTP1, SRD5A2, CYP2E1, CREB1, GFAP, CCL3, CAMK4, TPO, CYP1A1, CYP1B1, AHR, MUC1, CRYZ, TRPC3, TRPV3, ENOX2, GNRH1, HDC, HSD17B1, PNP, PDXP, CBS, GCLM, GSR, CTSB, SERPINA1, TNFSF13B, MMP3, METAP2, AHCY, CBR1, GSTA1, ADH7, GSTZ1, PFKFB4, GNPDA1, PFKM, WARS2, MPG, GALT, NPL, RBKS, G6PD, GAPDH, UMPS, ACAA1, ASRGL1, RTCA, HARS1, PDXK, EPHX2, FDXR, ALAD, PGLS, CBR4, ALDOA, UROD, APRT, RNASE1, ADH4, PNMT, ANXA3, CPA1, GM2A, GABRB1, NDUFS8, LCMT2, NDUFS3, NDUFS1, GABRB3, ALDH1B1, DMPK, HAGH, DHRS11, GSTA5, DCXR, CHDH, PHOSPHO1, RDH5, BHMT, TGM7, EGLN1, BHMT2, SIRT5, TRAPPC4, GSTM4, ACY1, RDH13, MCAT, GSTA2, HDAC8, PYCR1, ACADSB, PPIH, PRDX2, OAZ3, FKBP1A, GAMT, GLUL, PSMA1, KYAT1, FOLH1, MMUT, GCH1, PGD, PPOX, ASL, GUSB, CPB1, PLPBP, ATP5F1A, UQCRC1, PYCR2, NDUFV1, ALDH18A1, PCCA, ALDH9A1, ALDH4A1, AADAT, MGMT, SULT2B1, ALPP, C8G, PFKFB1, ARF4, COX5A, SPR, RDH11, HSD17B8, RDH8, SLC25A15, AMT, GALNS, GGT5, ACE, SELP, HS3ST3A1, GBA2, TREH, CDK2, FASLG, CYP19A1, CSF2, EGFR, BCL2L1, TOP1, MDM2, MCL1, BIRC5, TOP2A, CD40LG, PTGES, NUF2, ADCY2, MET, PRKCG, PRKCZ, CYP4B1, CYP2D6, SELE, VCAM1, GABBR1, F11R, JAK2, MT2A, SLC2A1, PECAM1, BMP4, SFTPB, PCDH12, NCF1, OLR1, SIRT1, MT-ND6, HSD3B2, HSD3B1, POR, HAS2, C5AR1, FCER2, ITGB2, TBXA2R, PSMD3, LDLR, BAD, MTTP, PLB1, UGT1A1, SREBF1, ABCC1, ADIPOQ, SOAT2, AKR1C1, CES1, HSD11B2, CTNNA1, JUP, RASGRF2, RAF1, MAPK7, CHKB, STAT3, CCL11, CYP11B2, FOSL2, CDK6, ATF2, MAPK8IP2, PTPN6, INPPL1, LITAF, CCND2, EGF, ELK1, ODC1, HSPA5, ACACA, CCL2, HSPB1, SULT1E1, PLAT, THBD, IL1A, NFE2L2, PARP1, COL3A1, CXCL11, CXCL2, DCAF5, CHEK2, CLDN4, HSF1, CXCL10, CHUK, SPP1, RUNX2, RASSF1, E2F1, E2F2, IGFBP3, IGF2, IRF1, ERBB3, PCOLCE, NPEPPS, HK2, NKX3-1, RASA1, CDC37, PDE10A, B3GAT1, CDC25C, APAF1, PLK1, SFN, CRAT, SLC38A3, DRD4, CCL16, SREBF2, COL7A1, G6PC1, CHI3L1, HK1, ACSL1, ACSL4, KLF7

S3. 559 terms of GO and KEGG Pathway enrichment.

Description
Pathways in cancer
Kaposi sarcoma-associated herpesvirus infection
Human cytomegalovirus infection
Human papillomavirus infection
Alzheimer disease
Small cell lung cancer
response to decreased oxygen levels
response to oxygen levels
cellular response to decreased oxygen levels
cellular response to oxygen levels
response to tumor necrosis factor
cellular response to organonitrogen compound
behavior
learning or memory
response to hypoxia
cellular response to nitrogen compound
MicroRNAs in cancer
cellular response to hypoxia
response to UV
response to light stimulus
cellular response to abiotic stimulus
cellular response to environmental stimulus
response to metal ion
response to radiation
regulation of smooth muscle cell proliferation
negative regulation of cell population proliferation

positive regulation of apoptotic process
positive regulation of programmed cell death
positive regulation of cell death
response to estradiol
response to glucocorticoid
negative regulation of cell cycle
response to corticosteroid
Pathways of neurodegeneration - multiple diseases
regulation of neuron apoptotic process
response to steroid hormone
response to xenobiotic stimulus
cellular response to organic cyclic compound
cell fate commitment
embryonic morphogenesis
plasma membrane protein complex
cytokine receptor binding
IL-17 signaling pathway
receptor ligand activity
signaling receptor activator activity
signaling receptor regulator activity
cytokine activity
Malaria
growth factor activity
JAK-STAT signaling pathway
Inflammatory bowel disease
positive regulation of MHC class II biosynthetic process
positive regulation of response to external stimulus
Leishmaniasis
positive regulation of cell adhesion
regulation of leukocyte migration
regulation of MHC class II biosynthetic process
positive regulation of leukocyte chemotaxis
T cell receptor signaling pathway
Cytokine-cytokine receptor interaction
mononuclear cell differentiation
positive regulation of cell-cell adhesion
regulation of leukocyte chemotaxis
regulation of autophagy
growth factor receptor binding
positive regulation of chemotaxis
positive regulation of leukocyte migration
African trypanosomiasis
positive regulation of receptor signaling pathway via JAK-STAT
Allograft rejection
positive regulation of lymphocyte activation
positive regulation of receptor signaling pathway via STAT
regulation of tumor necrosis factor production
positive regulation of leukocyte activation

B cell activation
regulation of tumor necrosis factor superfamily cytokine production
Intestinal immune network for IgA production
positive regulation of peptidyl-tyrosine phosphorylation
positive regulation of cell activation
positive regulation of immunoglobulin production
regulation of macrophage activation
regulation of chemotaxis
regulation of DNA metabolic process
positive regulation of tyrosine phosphorylation of STAT protein
positive regulation of T cell activation
positive regulation of immune effector process
regulation of immunoglobulin production
regulation of receptor signaling pathway via JAK-STAT
regulation of peptidyl-tyrosine phosphorylation
positive regulation of leukocyte cell-cell adhesion
lymphocyte differentiation
regulation of tyrosine phosphorylation of STAT protein
regulation of receptor signaling pathway via STAT
positive regulation of DNA metabolic process
regulation of chemokine production
Th17 cell differentiation
negative regulation of cytokine production
regulation of cytokine production involved in immune response
B cell differentiation
regulation of interleukin-1 production
positive regulation of lymphocyte differentiation
positive regulation of production of molecular mediator of immune response
myeloid leukocyte activation
positive regulation of B cell activation
T cell differentiation
regulation of interleukin-6 production
regulation of lymphocyte mediated immunity
regulation of adaptive immune response based on somatic recombination of immune receptors built from immunoglobulin superfamily domains
positive regulation of leukocyte differentiation
positive regulation of hemopoiesis
regulation of production of molecular mediator of immune response
leukocyte activation involved in immune response
cell activation involved in immune response
regulation of adaptive immune response
positive regulation of immune response
regulation of lymphocyte differentiation
regulation of leukocyte mediated immunity
immune effector process
regulation of vesicle-mediated transport
regulation of peptidyl-serine phosphorylation
positive regulation of peptidyl-serine phosphorylation
regulation of cell development

positive regulation of protein phosphorylation
regulation of nervous system development
regulation of neurogenesis
negative regulation of cell differentiation
positive regulation of neurogenesis
positive regulation of nervous system development
positive regulation of cell development
positive regulation of binding
positive regulation of cellular component biogenesis
positive regulation of cell migration
positive regulation of cell motility
positive regulation of locomotion
HIF-1 signaling pathway
PI3K-Akt signaling pathway
Insulin resistance
regulation of extrinsic apoptotic signaling pathway
Diabetic cardiomyopathy
negative regulation of extrinsic apoptotic signaling pathway
Sphingolipid signaling pathway
regulation of macroautophagy
cGMP-PKG signaling pathway
negative regulation of cell migration
negative regulation of cell motility
negative regulation of locomotion
regulation of apoptotic signaling pathway
negative regulation of apoptotic signaling pathway
regulation of anatomical structure size
response to fluid shear stress
regulation of metal ion transport
vascular process in circulatory system
caveola
membrane raft
membrane microdomain
regulation of ion transport
response to heat
plasma membrane raft
blood circulation
regulation of tube diameter
blood vessel diameter maintenance
regulation of tube size
female gamete generation
response to temperature stimulus
circulatory system process
regulation of calcium ion transport
ossification
gamete generation
regulation of T cell activation
regulation of cell activation

regulation of cell-cell adhesion
regulation of lymphocyte activation
regulation of T cell proliferation
regulation of leukocyte cell-cell adhesion
regulation of leukocyte activation
negative regulation of cell-cell adhesion
negative regulation of immune system process
regulation of cellular catabolic process
regulation of lymphocyte proliferation
regulation of mononuclear cell proliferation
regulation of leukocyte proliferation
negative regulation of cell adhesion
negative regulation of leukocyte cell-cell adhesion
Tuberculosis
regulation of B cell proliferation
regulation of B cell activation
negative regulation of cell activation
negative regulation of B cell proliferation
Toxoplasmosis
negative regulation of T cell activation
negative regulation of B cell activation
regulation of protein serine/threonine kinase activity
negative regulation of lymphocyte activation
negative regulation of leukocyte activation
negative regulation of protein kinase activity
negative regulation of kinase activity
negative regulation of T cell proliferation
negative regulation of transferase activity
negative regulation of lymphocyte proliferation
negative regulation of mononuclear cell proliferation
PD-L1 expression and PD-1 checkpoint pathway in cancer
negative regulation of leukocyte proliferation
negative regulation of protein phosphorylation
glial cell development
limbic system development
regulation of cyclin-dependent protein serine/threonine kinase activity
regulation of cyclin-dependent protein kinase activity
negative regulation of phosphorylation
negative regulation of protein serine/threonine kinase activity
protease binding
negative regulation of phosphate metabolic process
negative regulation of phosphorus metabolic process
pallium development
negative regulation of mitotic cell cycle
telencephalon development
rhythmic process
positive regulation of proteolysis
forebrain development

brain development
AGE-RAGE signaling pathway in diabetic complications
response to lipopolysaccharide
response to molecule of bacterial origin
Amoebiasis
Alcoholic liver disease
Non-alcoholic fatty liver disease
Influenza A
response to bacterium
response to hormone
Lipid and atherosclerosis
Pertussis
Salmonella infection
homeostasis of number of cells
Rheumatoid arthritis
cellular response to chemical stress
TNF signaling pathway
response to oxidative stress
Cellular senescence
Hepatitis B
leukocyte apoptotic process
response to reactive oxygen species
Legionellosis
response to inorganic substance
leukocyte homeostasis
Toll-like receptor signaling pathway
cytokine-mediated signaling pathway
cellular response to reactive oxygen species
Measles
positive regulation of cellular amide metabolic process
Herpes simplex virus 1 infection
Pathogenic Escherichia coli infection
regulation of cellular amide metabolic process
Epstein-Barr virus infection
cellular response to oxidative stress
Human T-cell leukemia virus 1 infection
cellular response to cytokine stimulus
humoral immune response
positive regulation of DNA-binding transcription factor activity
regulation of DNA-binding transcription factor activity
FoxO signaling pathway
gland development
Prostate cancer
regulation of epithelial cell proliferation
glial cell differentiation
regulation of myelination
negative regulation of cell development
Focal adhesion

positive regulation of MAPK cascade
Endometrial cancer
regulation of mitotic cell cycle
negative regulation of protein modification process
gliogenesis
regulation of MAPK cascade
cell population proliferation
regulation of epithelial to mesenchymal transition
Autophagy - animal
negative regulation of neurogenesis
Breast cancer
negative regulation of nervous system development
mTOR signaling pathway
Hepatocellular carcinoma
regulation of protein modification by small protein conjugation or removal
regulation of cell cycle process
cell surface receptor signaling pathway involved in cell-cell signaling
chemical synaptic transmission
anterograde trans-synaptic signaling
trans-synaptic signaling
synaptic signaling
carbohydrate metabolic process
protein kinase binding
kinase binding
regulation of epithelial cell migration
angiogenesis
tube morphogenesis
blood vessel morphogenesis
negative regulation of catalytic activity
blood vessel development
vasculature development
Fluid shear stress and atherosclerosis
positive regulation of epithelial cell migration
VEGF signaling pathway
endothelial cell migration
positive regulation of blood vessel endothelial cell migration
EGFR tyrosine kinase inhibitor resistance
epithelial cell migration
epithelium migration
tissue migration
positive regulation of endothelial cell migration
regulation of blood vessel endothelial cell migration
ameboidal-type cell migration
regulation of endothelial cell migration
negative regulation of cysteine-type endopeptidase activity involved in apoptotic process
negative regulation of cysteine-type endopeptidase activity
negative regulation of hydrolase activity
Relaxin signaling pathway

regulation of cysteine-type endopeptidase activity involved in apoptotic process
Chemical carcinogenesis - reactive oxygen species
positive regulation of protein serine/threonine kinase activity
regulation of cysteine-type endopeptidase activity
negative regulation of endopeptidase activity
negative regulation of peptidase activity
regulation of endopeptidase activity
regulation of peptidase activity
positive regulation of hydrolase activity
protein homodimerization activity
negative regulation of cellular component organization
inflammatory response
positive regulation of transferase activity
regulation of acute inflammatory response
positive regulation of nucleotide metabolic process
positive regulation of purine nucleotide metabolic process
regulation of protein kinase activity
regulation of inflammatory response
positive regulation of cellular catabolic process
regulation of kinase activity
positive regulation of cytokine production
positive regulation of catabolic process
regulation of purine nucleotide metabolic process
regulation of nucleotide metabolic process
regulation of defense response
regulation of generation of precursor metabolites and energy
regulation of immune effector process
positive regulation of ATP metabolic process
regulation of neuroinflammatory response
positive regulation of protein kinase activity
negative regulation of response to external stimulus
positive regulation of kinase activity
regulation of ATP metabolic process
alpha-beta T cell activation
negative regulation of immune effector process
activation of protein kinase activity
positive regulation of inflammatory response
regulation of protein transport
regulation of peptide hormone secretion
regulation of peptide secretion
negative regulation of inflammatory response
regulation of peptide transport
regulation of establishment of protein localization
negative regulation of immune response
regulation of secretion by cell
regulation of secretion
regulation of hormone secretion
regulation of protein secretion

negative regulation of defense response
positive regulation of secretion by cell
positive regulation of protein transport
positive regulation of secretion
positive regulation of defense response
positive regulation of establishment of protein localization
endoplasmic reticulum lumen
regulation of hormone levels
positive regulation of small molecule metabolic process
regulation of small molecule metabolic process
negative regulation of oxidative stress-induced cell death
regulation of cellular response to stress
regulation of nitric oxide biosynthetic process
regulation of proteolysis
regulation of nitric oxide metabolic process
regulation of oxidative stress-induced cell death
positive regulation of protein localization to nucleus
positive regulation of lipid biosynthetic process
regulation of cellular response to oxidative stress
positive regulation of smooth muscle cell proliferation
regulation of response to oxidative stress
C-type lectin receptor signaling pathway
regulation of protein localization to nucleus
negative regulation of proteolysis
positive regulation of monooxygenase activity
regulation of fat cell differentiation
positive regulation of lipid metabolic process
positive regulation of nitric oxide biosynthetic process
positive regulation of nitric oxide metabolic process
regulation of lipid biosynthetic process
negative regulation of transport
regulation of monooxygenase activity
Regulation of lipolysis in adipocytes
positive regulation of oxidoreductase activity
positive regulation of fat cell differentiation
positive regulation of carbohydrate metabolic process
regulation of fatty acid metabolic process
negative regulation of intrinsic apoptotic signaling pathway
regulation of protein localization to plasma membrane
regulation of lipid metabolic process
regulation of oxidoreductase activity
regulation of protein catabolic process
response to peptide hormone
regulation of protein localization to cell periphery
regulation of cellular ketone metabolic process
placenta development
response to peptide
positive regulation of protein localization

regulation of protein localization to membrane
regulation of intrinsic apoptotic signaling pathway
regulation of carbohydrate metabolic process
negative regulation of intracellular signal transduction
regulation of cellular macromolecule biosynthetic process
glucose homeostasis
carbohydrate homeostasis
response to insulin
positive regulation of transmembrane transport
negative regulation of cellular catabolic process
negative regulation of catabolic process
positive regulation of cell cycle
regulation of transmembrane transport
leukocyte activation
cell activation
lymphocyte activation
T cell activation
Chagas disease
regulation of angiogenesis
regulation of vasculature development
cellular response to lipopolysaccharide
cellular response to molecule of bacterial origin
cellular response to biotic stimulus
Yersinia infection
positive regulation of angiogenesis
positive regulation of vasculature development
cell chemotaxis
chemotaxis
taxis
cellular response to lipid
locomotion
Viral protein interaction with cytokine and cytokine receptor
leukocyte chemotaxis
leukocyte migration
cellular response to growth factor stimulus
response to growth factor
reproductive structure development
reproductive system development
chordate embryonic development
embryo development ending in birth or egg hatching
in utero embryonic development
Hepatitis C
embryonic organ development
Proteoglycans in cancer
Rap1 signaling pathway
positive regulation of epithelial cell proliferation
heart development
tissue morphogenesis

muscle cell differentiation
sex differentiation
Colorectal cancer
female gonad development
development of primary female sexual characteristics
positive regulation of endothelial cell proliferation
female sex differentiation
tube formation
morphogenesis of an epithelium
morphogenesis of a branching epithelium
morphogenesis of a branching structure
muscle structure development
lung development
regulation of endothelial cell proliferation
respiratory tube development
respiratory system development
striated muscle cell differentiation
epithelial cell differentiation
gonad development
development of primary sexual characteristics
epithelial tube morphogenesis
cell-cell junction
protein catabolic process
regulation of neuron death
regulation of binding
regulation of protein localization to cell surface
negative regulation of neuron death
regulation of protein binding
hemopoiesis
hematopoietic or lymphoid organ development
leukocyte differentiation
immune system development
myeloid cell differentiation
myeloid leukocyte differentiation
negative regulation of epithelial cell differentiation
regulation of osteoclast differentiation
positive regulation of neuron death
apoptotic signaling pathway
regulation of leukocyte differentiation
regulation of myeloid leukocyte differentiation
regulation of hemopoiesis
regulation of epithelial cell differentiation
regulation of myeloid cell differentiation
transmembrane receptor protein tyrosine kinase signaling pathway
MAPK signaling pathway
enzyme-linked receptor protein signaling pathway
neuron projection development
positive regulation of cell growth

cell morphogenesis involved in differentiation
Ras signaling pathway
positive regulation of growth
regulation of growth
regulation of plasma membrane bounded cell projection organization
regulation of cell projection organization
cell morphogenesis
regulation of developmental growth
axon development
regulation of cell growth
regulation of neuron projection development
regulation of axonogenesis
neuron projection morphogenesis
plasma membrane bounded cell projection morphogenesis
cell projection morphogenesis
positive regulation of developmental growth
cell part morphogenesis
regulation of cell size
regulation of cell junction assembly
cellular component morphogenesis
developmental maturation
cell junction assembly
synapse organization
urogenital system development
positive regulation of cell projection organization
axonogenesis
regulation of cellular component size
growth
developmental growth
cell morphogenesis involved in neuron differentiation
cell junction organization
negative regulation of smooth muscle cell proliferation
regulation of nervous system process
regulation of response to wounding
regulation of system process
tissue homeostasis
anatomical structure homeostasis
multicellular organismal homeostasis
maintenance of blood-brain barrier
wound healing
myeloid cell homeostasis
response to wounding
regulation of body fluid levels
secretion
cognition
regulation of synapse organization
regulation of synapse structure or activity
positive regulation of synaptic transmission

modulation of chemical synaptic transmission
regulation of trans-synaptic signaling
regulation of synaptic plasticity
Phospholipase D signaling pathway
second-messenger-mediated signaling
